# Supplementary material for: Species-aware DNA language models capture regulatory elements and their evolution
Source: Genome Biol. 2024 Apr 2;25:83. doi: 10.1186/s13059-024-03221-x (PMC10985990; doi:10.1186/s13059-024-03221-x)
Supplement: Supplementary file 1 — Additional file 1. PDF containing supplementary figures and analyses cited in the main text. [file 13059_2024_3221_MOESM1_ESM.pdf]

Additional file 1

Supplementary Figures
Motif Discovery
- Modisco - 3’
- Modisco - 5’
- Streme - 3’
- Streme - 5’

Species Token Choice

Supplementary Figures

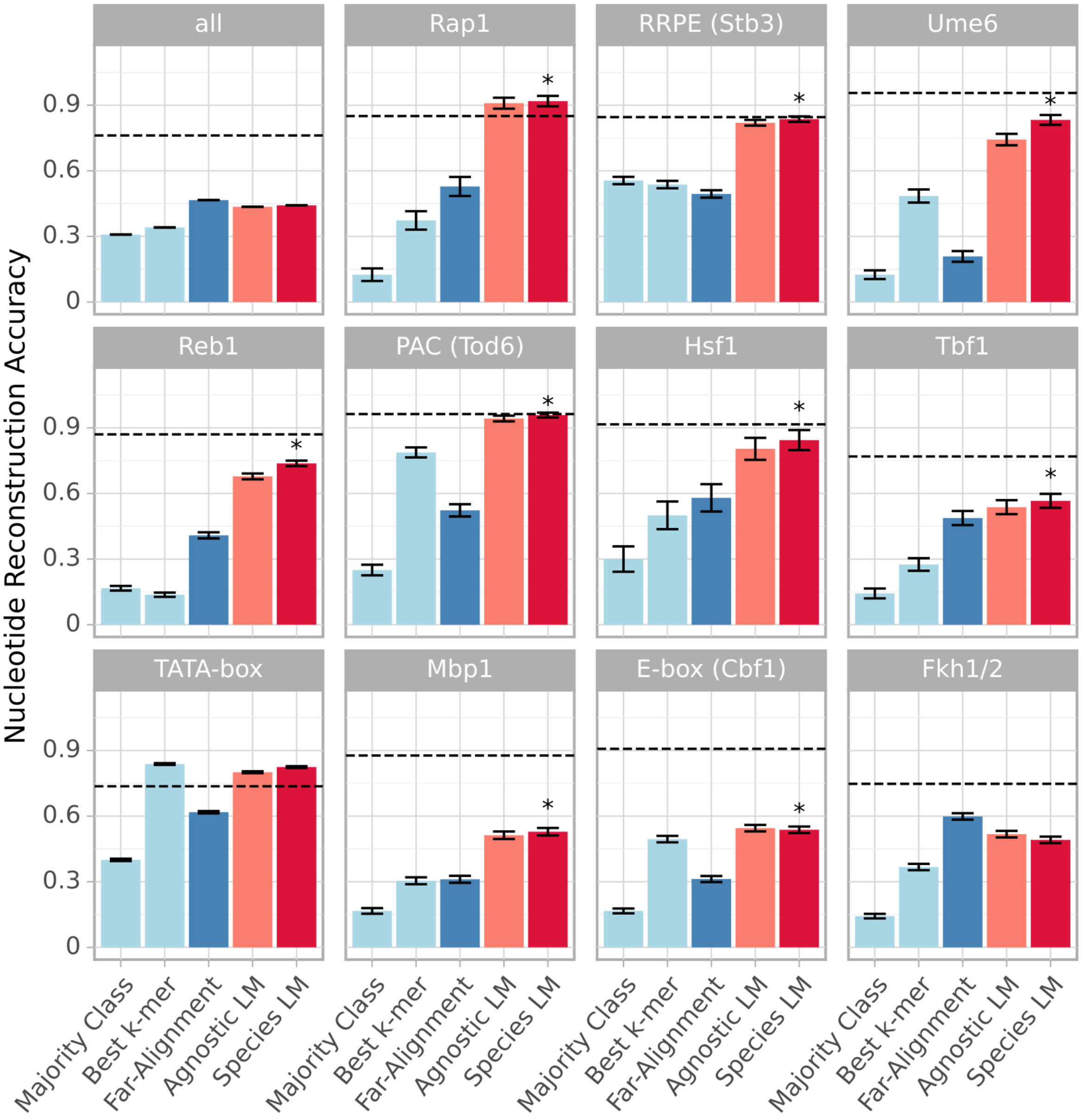

Fig S1: Language models reconstruct likely regulatory sequences in the 5' regions of a held-out species. Reconstruction accuracy for nucleotides within instances of Transcription Factor consensus motifs and across all nucleotides in *S. cerevisiae* 5' regions. We compare the agnostic and species 5' LM to a variety of baselines. Dashed line represents the accuracy achieved by the intra-genus alignment. Star indicates that the species LM significantly (*P* < 0.05, binomial test) outperforms the best baseline. In many motifs the LMs clearly outperform the baselines. Note that particularly in shorter motifs the reconstruction given here is likely an underestimate, since we consider all matches to the consensus even if many are likely non-functional in-vivo due to their context.

The used consensus motifs are:

- **Rap1**: CAYCCRTACAY
- **RRPE**: AATTTTTCA
- **Ume6**: TAGCCGCC
- **Reb1**: MGGGTAA
- **PAC**: GMGATGAGMT
- **Hsf1**: GAANNTTCTRGAA
- **Tbf1**: ARCCCTAA
- **TATA-box**: TATAWAWR
- **Mbp1**: ACGCGT
- **E-box**: CACGTG
- **Fkh1/2**: GTAAACA

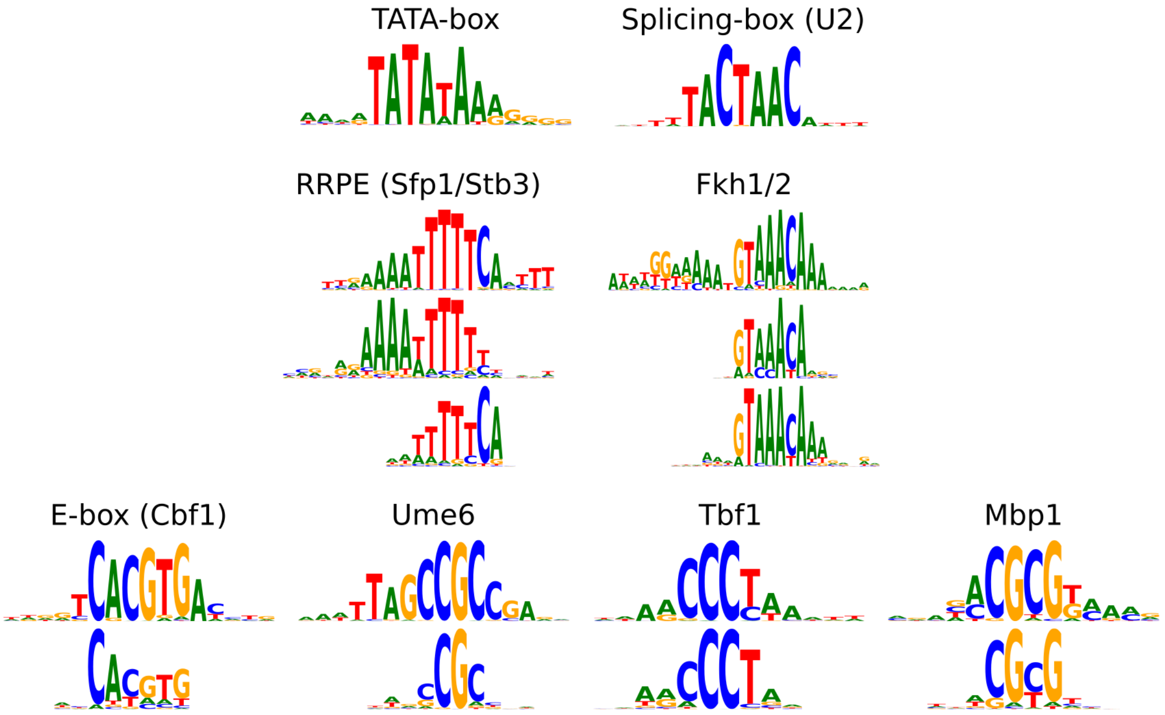

Fig S2: Language models recover known binding motifs. Known transcription factor binding motifs (as well as the splicing motif recognized by the U2-like snRNA) recovered by applying Modisco clustering to the 5' species LM reconstructions. Where possible, the Modisco motif (above) is manually matched to the respective high-confidence PWM from the Yetfasco database (below). We note that the Yetfasco Ume6 motif is derived from in-vitro data, in Chip-exo data the binding preference of Ume6 is much more resemblant of the motif found by Modisco.

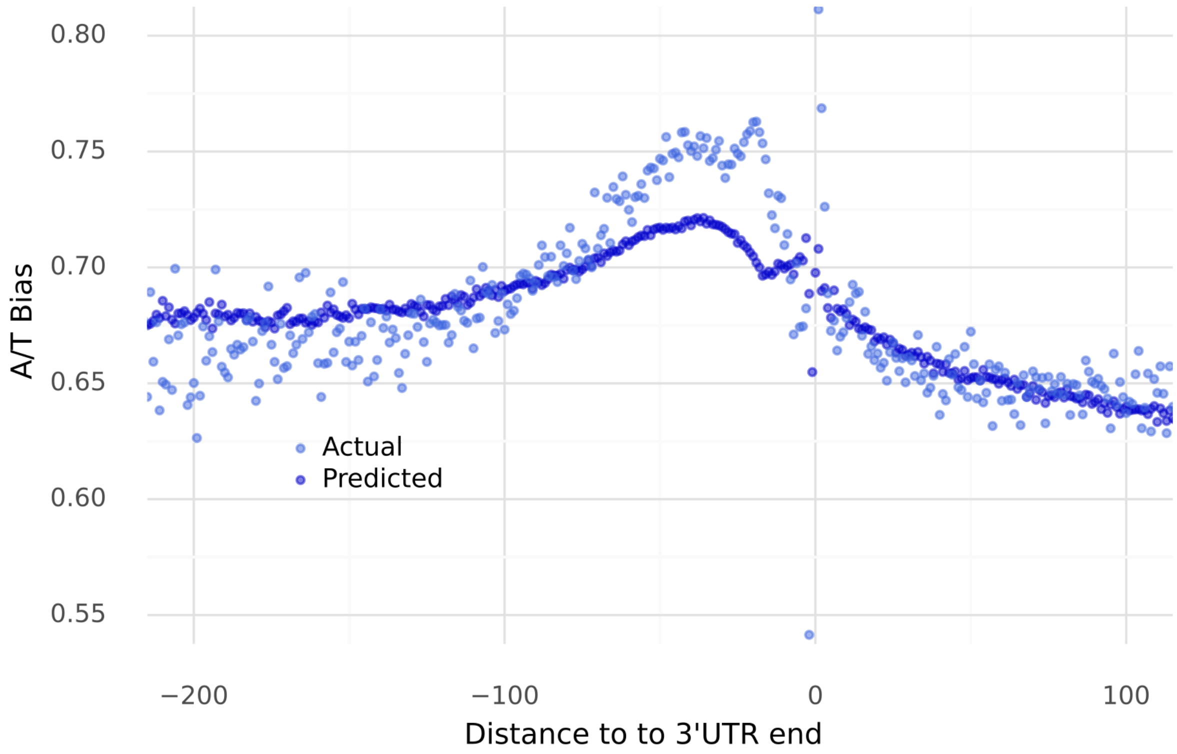

Fig S3: The model mimics the elevated AT-bias 5’ of the polyadenylation site. Actual and predicted (by the 3’ species LM) nucleotide biases as function of the distance to the end of the 3’ UTR. The model keeps track of local variations in AT bias.

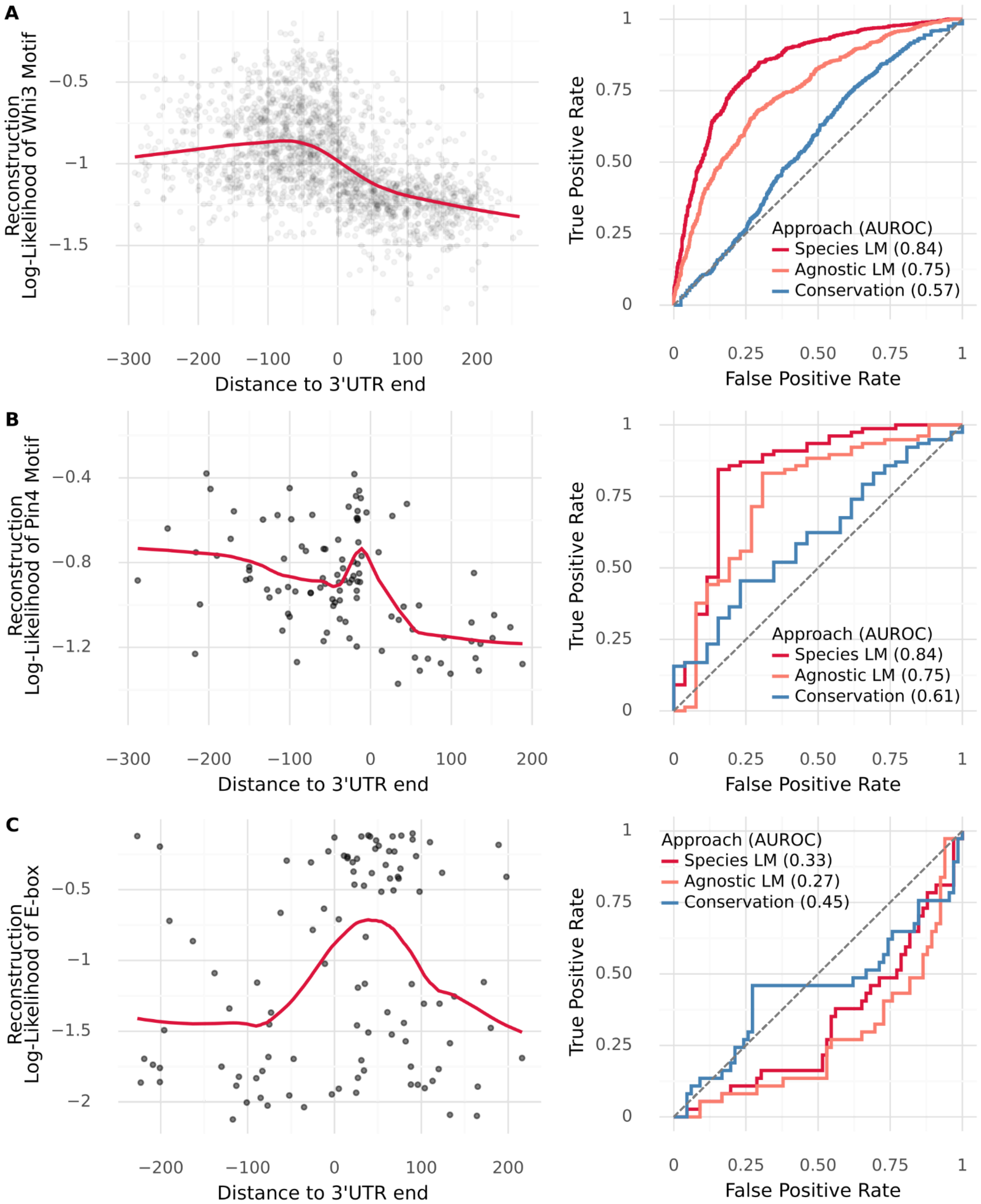

Fig S4: Reconstruction of motifs depends on context. A) Left: Reconstruction fidelity (log-likelihood of the observed nucleotides according to the 3' Species LM) of instances of the Whi3 motif (TGCAT), as function of the distance to the end of the annotated 3'UTR. Right: ROC curve evaluating to what extent the reconstruction fidelity of our 3' LMs, as well as the phastCons conservation score, can serve as a predictor of whether a Puf3 motif instance is within or beyond the 3'UTR boundary. **B)** Same for Pin4 (TTTAATGA). **C)** Same for the E-box (CACGTG), a transcription factor binding motif. We expect TF motifs to be depleted in transcribed regions and indeed the reconstruction fidelity peaks after the end of the 3'UTR.

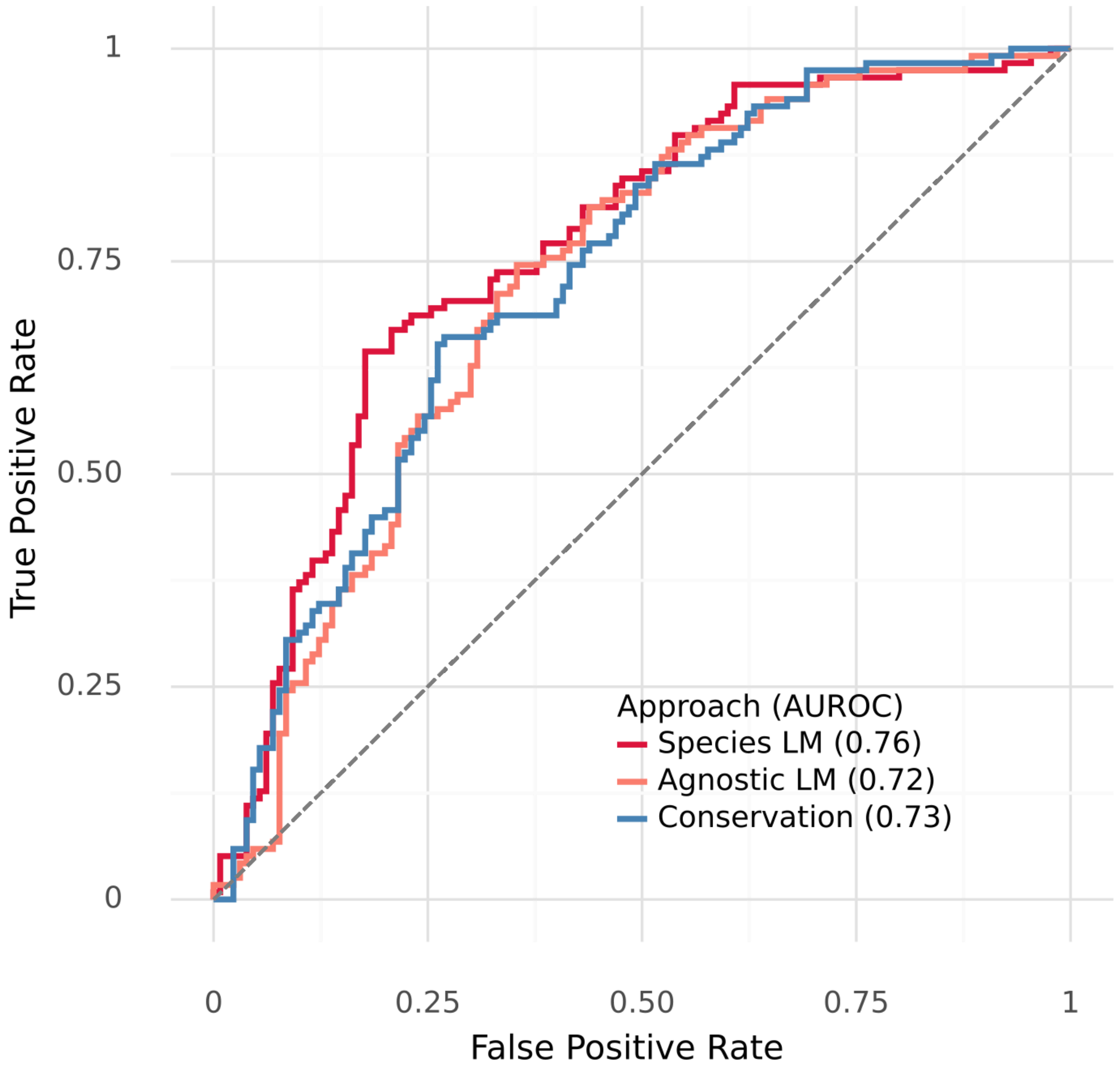

Fig S5: Reconstruction fidelity predicts whether a Puf3 motif instance is located 3' of a gene bound by Puf3p in-vivo. We use the reconstruction fidelity of a Puf3 motif (TGTAAATA) instance achieved by the 3' LMs as a classifier of whether there is experimental evidence that the upstream gene is bound by Puf3p. We also compare against using the phastCons conservation score, which in this case performs on par with the LMs.

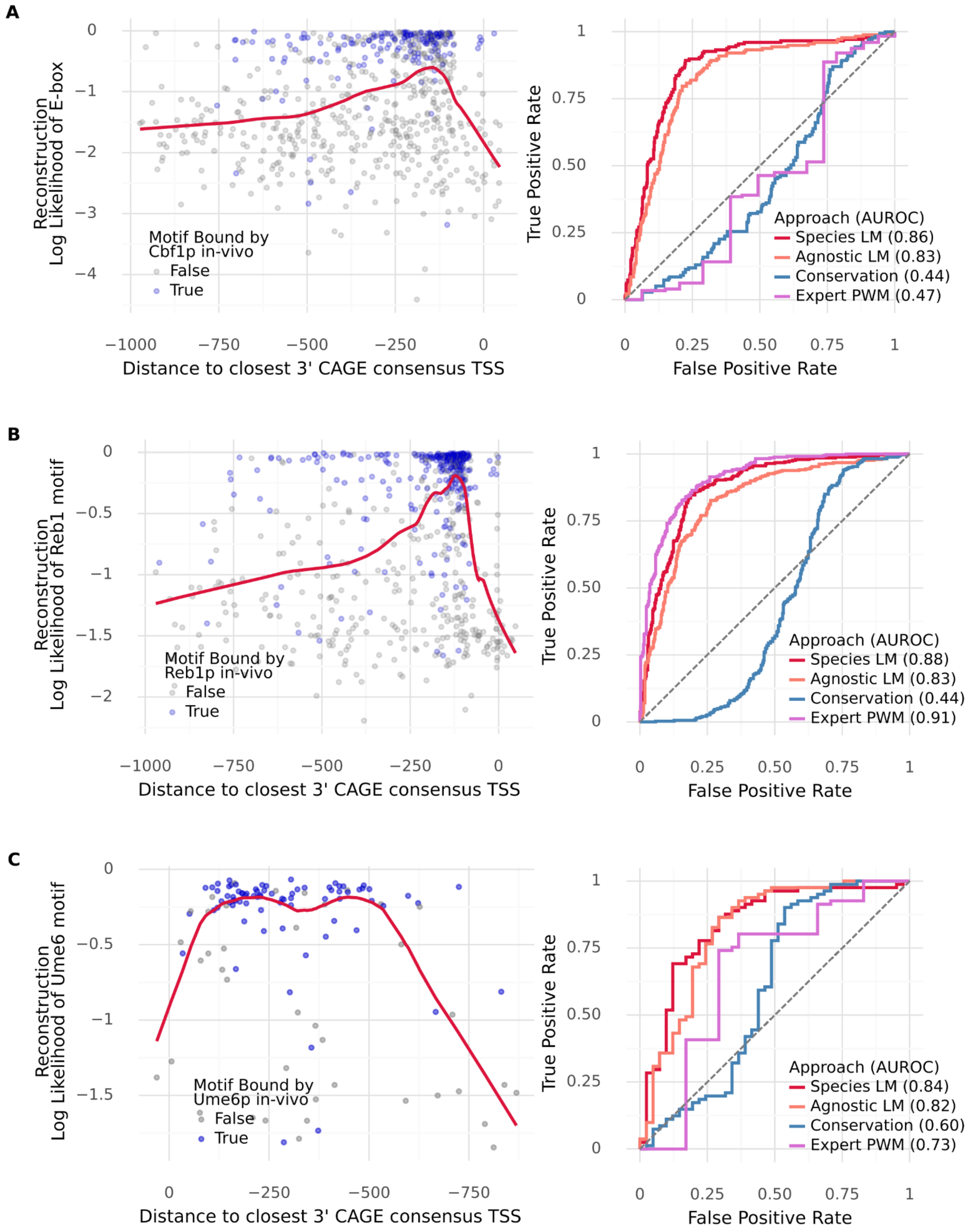

Fig S6: Reconstruction of TF motifs depends on context and predicts whether a motif instance will be bound in-vivo. A) Left: Reconstruction fidelity (log-likelihood of the observed nucleotides according to the 5' Species LM) of instances of the E-box (CACGTG), as function of the distance to the closest 3' TSS (imputed using CAGE data). Blue indicates that the motif instance was bound in-vivo according to Chip-exo data. Right: ROC curve evaluating to what extent the reconstruction fidelity of our 5' LMs, as well as the phastCons conservation score and an expert curated PWM, can serve as a predictor of whether a E-box motif instance is bound in-vivo by Cbf1p. **B)** Same for the Reb1 motif (MGGGTAA). **C)** Same for the Ume6 motif (TAGCCGCC).

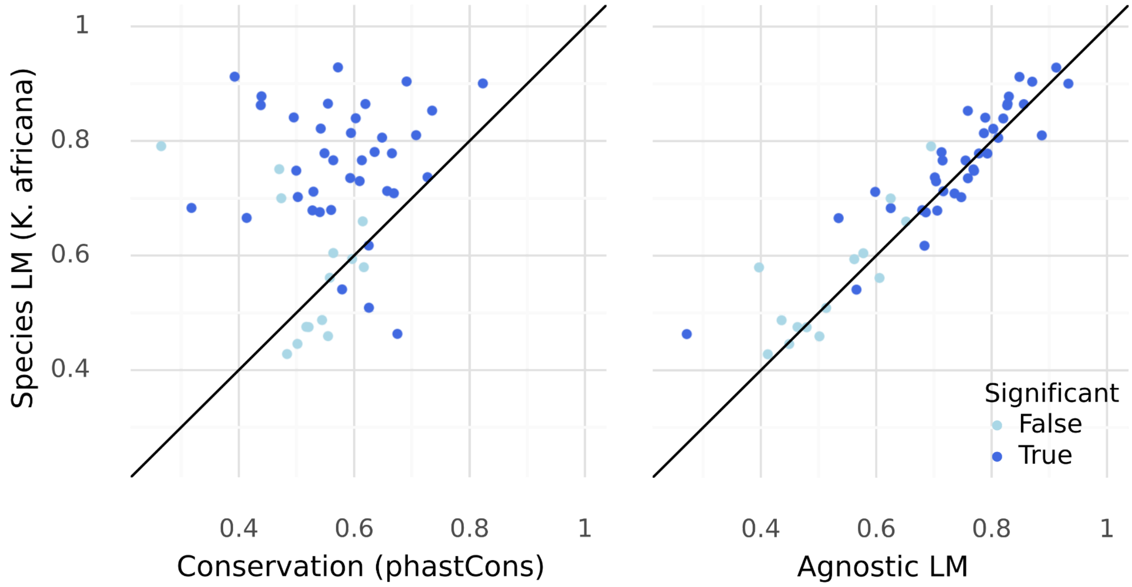

Fig S7: The predictiveness of the species LM reconstructions for in-vivo binding generalizes across a variety of yeast TF. We evaluated to what extent the reconstruction fidelity of our 5’ LMs, as well as the phastCons conservation score, can serve as a predictor of whether consensus motif instances of a variety of TF are bound in-vivo. For this we analyzed all yeast TF which (1) show more than 100 peaks in the Rossi et al. CHIP-exo dataset and (2) have a motif of at least medium confidence in the YeTFaSCo database. Altogether these are 51 TF. In the left panel, we compare the AUROC achieved per TF of the species LM (with *K. africana* as proxy token) with that achieved by the conservation score. Each point represents one TF. Dark blue color indicates that the AUROC achieved by at least one of the methods being compared is significantly different from random ($P < 0.05$, Mann-Whitney U). Altogether, the species LM decisively outperforms the phastCons score ($P = 1.3 \times 10^{-6}$, two-sided paired Wilcoxon test). The right panel compares the Species LM with the agnostic LM. The species LM slightly outperforms the agnostic LM ($P = 0.011$).

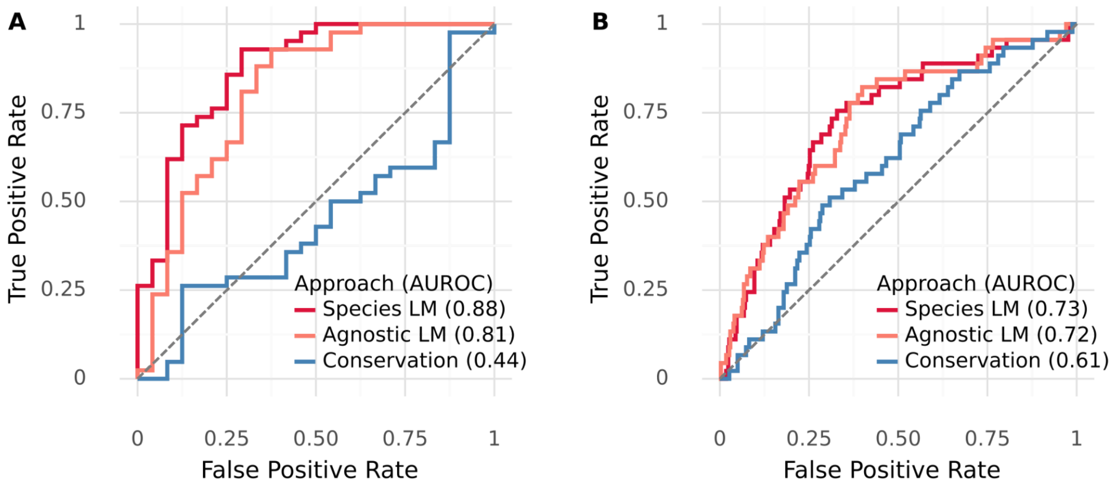

Fig S8: Reconstruction fidelity is predictive of the gene module. A) ROC curve evaluating to what extent the reconstruction fidelity of our 3' LMs, as well as the phastCons conservation score, can serve as a predictor of whether a Rap1 motif instance (CAYCCRTACAY) is located within 1kb 5' of a gene which forms part of the ribosomal protein (RP) module. **B)** ROC curve evaluating to what extent the reconstruction fidelity of our 3' LMs, as well as the phastCons conservation score, can serve as a predictor of whether a RRPE (Stb3) motif instance (AATTTTTCA) is located within 1kb 5' of a gene which forms part of the ribosome biogenesis module.

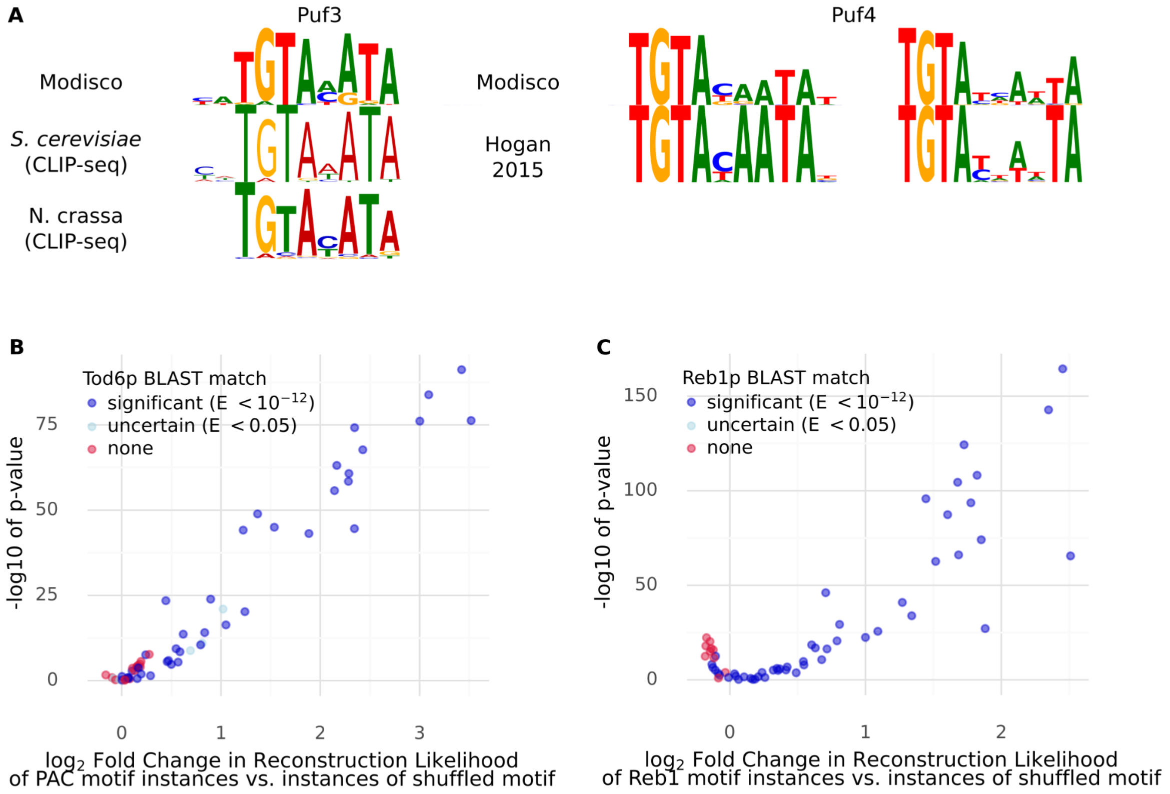

Fig S9: LMs can trace the presence and disappearance of motifs across species. A) Motifs recovered by Modisco clustering to the predictions of the 3' species LM on the 3' regions of all CBP3 homologues in our dataset. We recovered a Puf3 motif and two Puf4 motifs. As we are clustering across species, the recovered Puf3 motif appears to be a hybrid between the *S. cerevisiae* Puf3 motif (which has a preference for a C at -2 and an A in the middle) and the *N. crassa* Puf3 motif (which seemingly has a slight preference for C in the middle). **B)** We computed the reconstruction fidelity (log-likelihood) achieved by the species 5' LM for the *S. cerevisiae* consensus PAC motif instances and for instances matching shuffled versions of this motif in 60 fungal species. The difference in reconstruction between the true and shuffled motif instances, expressed as log2 fold change, is plotted against the -log10 p-value of this difference, computed using a Mann-Whitney-U test. We observe that in species which have no BLAST match to *S. cerevisiae* Tod6p, the reconstruction fidelity of the *S. cerevisiae* Tod6 motif is generally not much better than that of shuffled versions thereof. **C)** Same as B), but for Reb1.

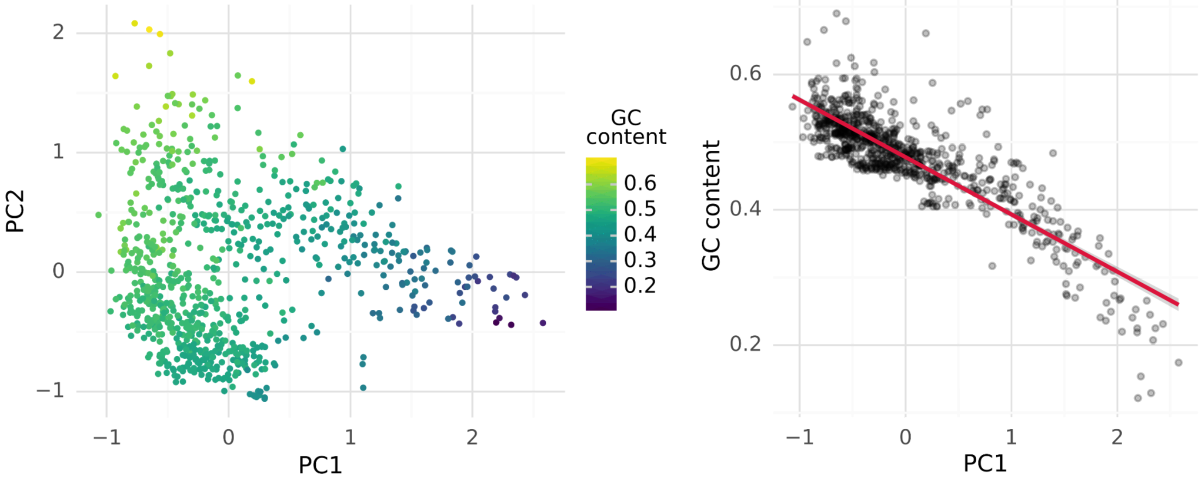

Fig S10: The first principal component of the species representation encodes the GC content. The left panel shows the first and second principal components of the species representation learned by the 5' species LM. Each point corresponds to a species in our training dataset. The brighter the color, the higher the GC content of the species' genome. Right panel: the first principal component, which explains ca. 10% of variance, is highly correlated (r = -0.86) with the GC content of the different fungal species.

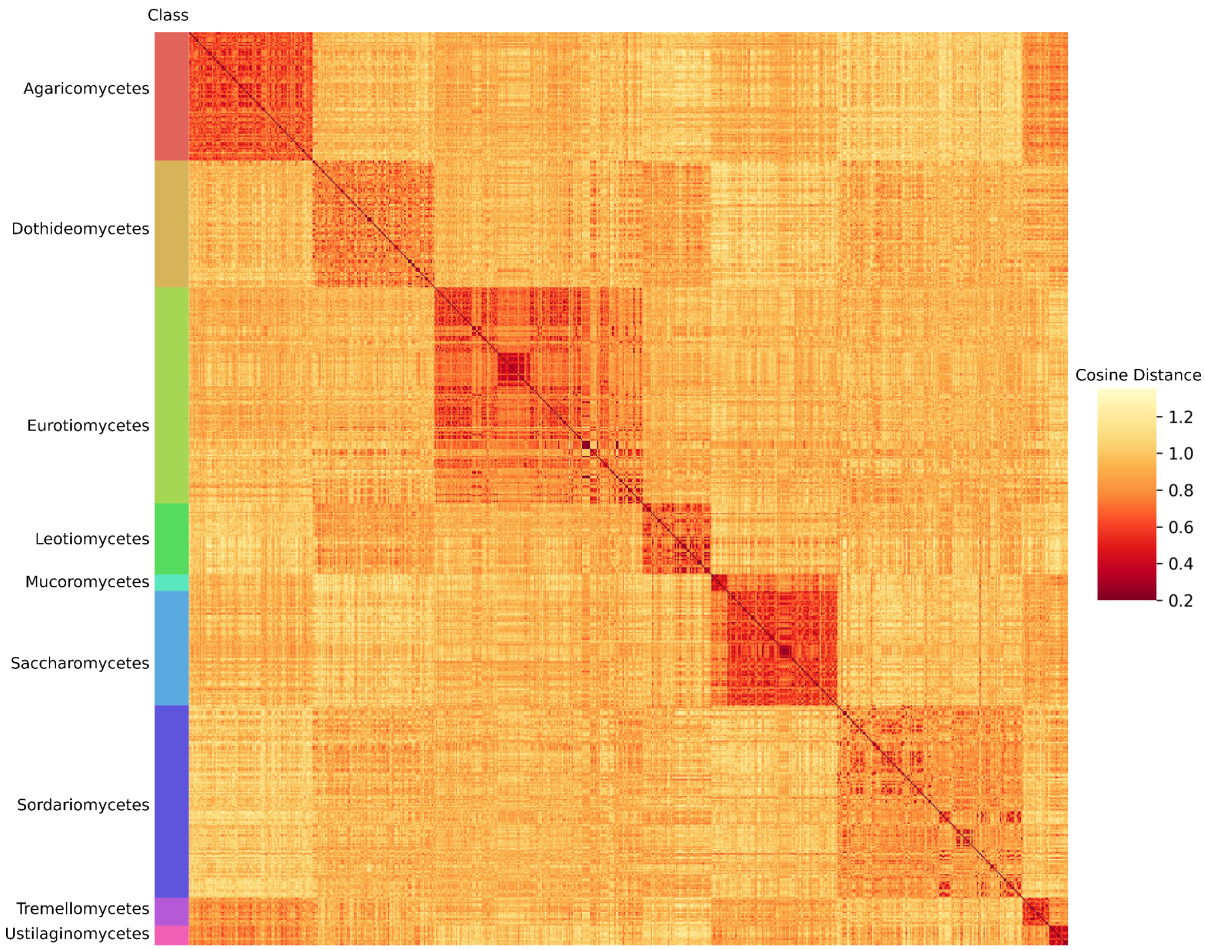

Fig S11: Cosine distances between learned species representations of the 5' species LM, annotated by the taxonomic class. Only classes with more than 10 members are shown.

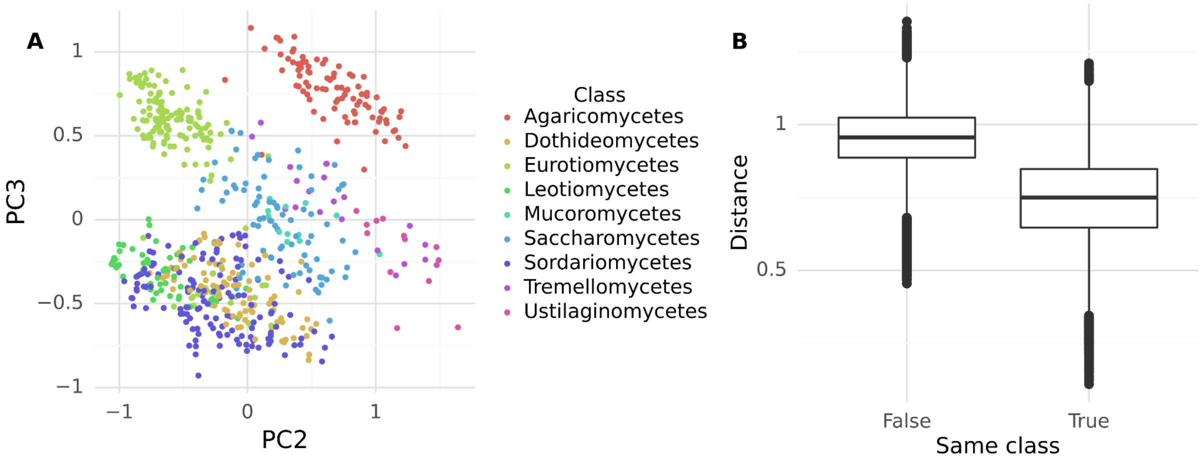

Fig S12: The species LM has learned to encode features in the species representation which partially reconstruct the fungal taxonomy. A) The figure shows the second (explains ~7% of the variance) and third (explains ~5% of the variance) principal components of the species representations learned by the 5' species LM. Each point corresponds to a species in our training dataset. Species are colored by their taxonomic class (only classes with more than 10 members are shown). We see that some classes form clearly separated clusters. If we apply *k*-means clustering ($k = 9$) to the first 16 principal components, which together explain half of the variance, and overlap the resulting clusters with the taxonomic classes, we receive an adjusted mutual information of 0.76. **B)** The cosine distances between representations within the same taxonomic class tends to be lower than the distances of representations corresponding to species from different taxonomic classes. The difference in distance is statistically significant ($P = 0.0009$, permutation test).

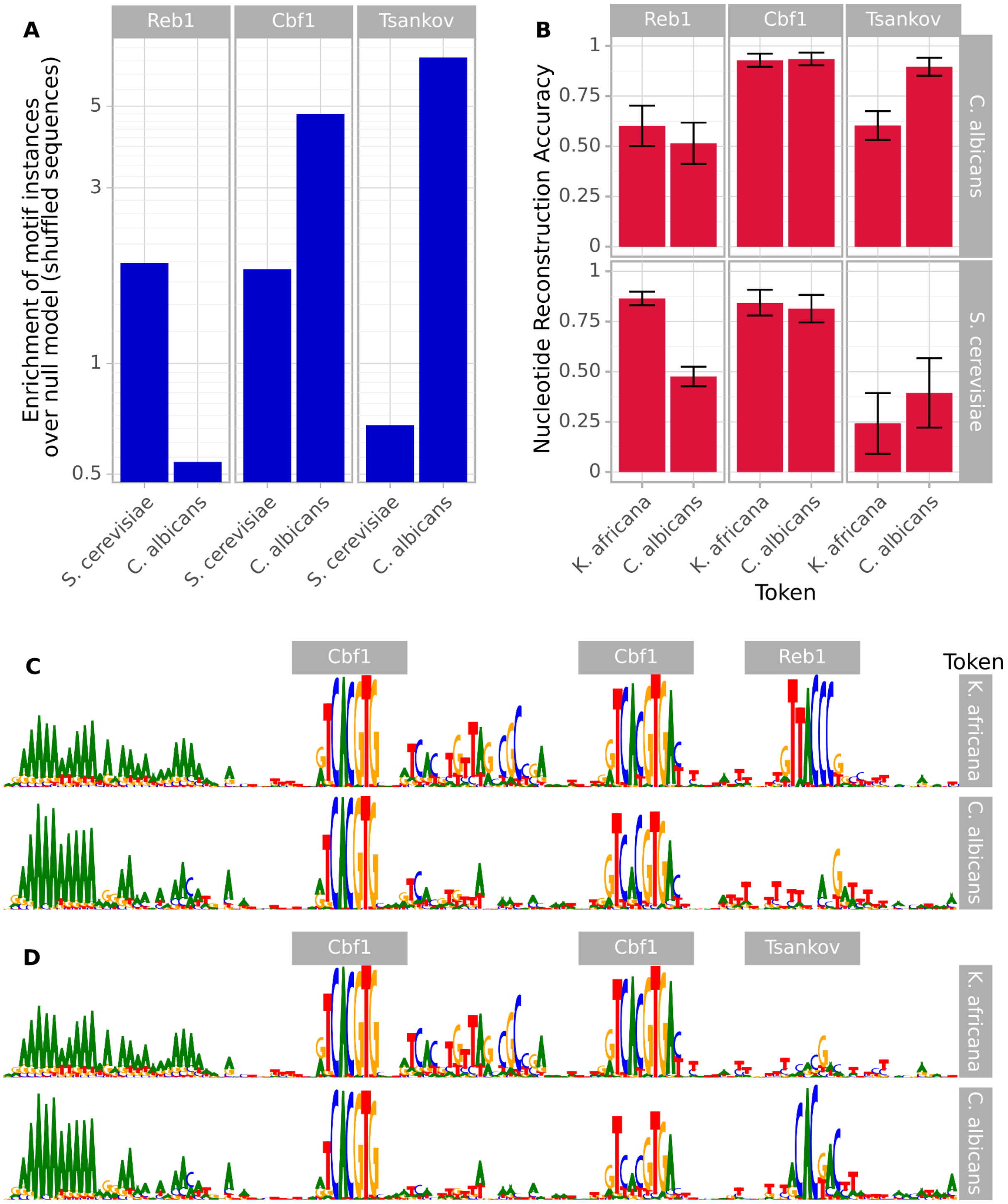

Fig S13: Swapping the species token causes the species LM to adjust its motif predictions. A) The number of occurrences of three known motifs - Reb1 (CGGGTAA), Cbf1 (tCACGTGa) and "Tsankov" (aCACGACc) - are compared to their expected occurrences in the *S. cerevisiae* and *C. albicans* 5' regions. Expected occurrences are computed by counting the motif occurrences in shuffled sequences, which preserves GC content but no higher-order features. We observe that Reb1 is enriched only in *S. cerevisiae*, Cbf1 is enriched in both species and the Tsankov motif is enriched only in *C. albicans*. **B)** Reconstruction accuracy achieved by the species LM, using two different species tokens, for motif instances in the *C. albicans* 5' regions (upper panels) and the *S. cerevisiae* 5' regions (lower panels). With the *K. africana* token (our proxy for *S. cerevisiae*), the species LM reconstructs the Reb1 motif better in both genomes. Conversely, with the *C. albicans* token, then the *C. albicans* specific Tsankov motif is reconstructed better in both species. Cbf1 is reconstructed regardless of token. **C)** Nucleotide predictions, scaled by information content, of the species LM for the promoter of *S. cerevisiae YDR510W*. With the *K. africana* token (upper panel), the Reb1 motif is reconstructed well but reconstruction collapses with the *C. albicans* token. **D)** In the same promoter, we *in-silico* replaced the Reb1 motif with the Tsankov motif. This is only reconstructed with the *C. albicans* token.

	S. cerevisiae	*S. pombe*	*S. japonicus*	*V. polyspora*	*D. hansenii*	*C. albicans*	*C. glabrata*	*K. lactis*
Best k-mer	0.081	0.146	0.129	0.218	0.078	0.125	0.181	0.115
Agnostic LM	0.317	0.242	0.219	0.410	0.269	0.301	0.425	0.310
Species LM	**0.366**	**0.341**	**0.286**	**0.437**	**0.321**	**0.327**	**0.456**	**0.342**

Table S1: Sequence representations of the species LM outperform other methods on microarray data of Thompson et al.
Performance (R^2) of linear models trained on embeddings from language models compared to best k-mer count regressions, where the best k from {3, 4, 5} is shown.

Model	*S. cerevisiae* RNA half-life, Cheng et al.	*S. pombe* RNA half-life, Eser et al.	*S. cerevisiae* MPRA, Shalem et al.	*S. cerevisiae* gene expression, Zrimec et al. 3'	*S. cerevisiae* gene expression, Zrimec et al. 5'	*S. cerevisiae* gene expression, Zrimec et al. 3' + 5'	Terminator MPRA, Yamanishi et al.	Promoter MPRA, Keren et al.
3-mer	0.580	0.433	0.276	0.046	0.055	0.079	0.020	0.007
4-mer	0.569	0.450	0.278	0.040	0.076	0.094	0.029	0.062
5-mer	0.532	0.400	0.337	0.017	0.080	0.073	0.024	0.230
SOTA	0.586	0.422		0.230	0.414	0.492	0.099	0.440
Nucleotide Transformer	0.530	0.404	0.413	0.104	0.135	0.208	0.045	0.272
Agnostic LM, yeast only, including sacc. genus	0.578	0.470	0.609	0.243				
Agnostic LM	0.578	0.479	0.532	0.240	0.365	0.438	0.071	0.352
Agnostic LM, including sacc. genus	0.580	0.476	0.530	0.276				
Species LM, yeast only, including sacc. genus	0.583	0.471	0.577	0.206				
Species LM	0.592	0.494	0.704	0.297	**0.492**	**0.550**	**0.108**	**0.454**
Species LM, including sacc. genus	**0.599**	**0.502**	**0.711**	**0.354**				

Table S2: Sequence representations of the species LM outperform other methods on a variety of downstream tasks.
Performance (R^2) of linear models trained on embeddings from language models compared to state-of-the-art models and 3-mer, 4-mer and 5-mer count regressions.

Motif Discovery

To assess how de-novo discovery of motifs from the language model using Modisco compares to traditional approaches, we compare to STREME, a popular and fast motif finding method. Below we have tabulated all Modisco and STREME results for *S. cerevisiae* 5’ and 3’ regions.

Overall, we find that Modisco clustering on the LM performs similarly to STREME. Specifically, with Modisco we find 31 hits for 3’ regions, of which we could match 6 (~19%) to known motifs and 12 are easily identified as simple repeats. For the 5’ regions, we get 51 hits, whereby 18 (~35%) correspond to known motifs and 13 correspond to simple repeats.

With STREME, we find 15 hits in 3’ regions (using default settings except that we use RNA mode and a minimum width of 5, as the Whi3 motif is only five nucleotides long), whereby 3 (~20%) correspond to known motifs and 5 correspond to simple repeats. For the 5’ regions, STREME (default settings) get 39 hits, whereby 9 (~23%) correspond to known motifs and 5 correspond to simple repeats.

Modisco - 3’

Name	Fwd	Rev	Match
3'_modisco_00	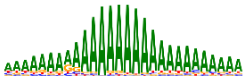	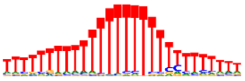	Repeat
3'_modisco_01	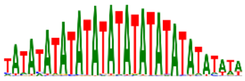	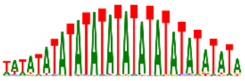	Repeat
3'_modisco_02	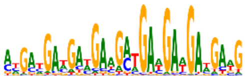	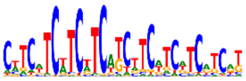	Repeat
3'_modisco_03	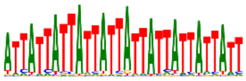	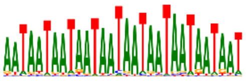	Repeat
3'_modisco_04, TATA-box	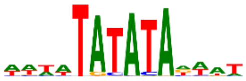	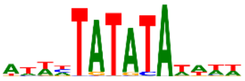	**TATAWAWR**
3'_modisco_05	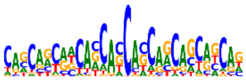	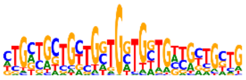	Repeat
3'_modisco_06, Puf3	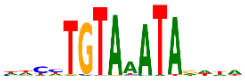	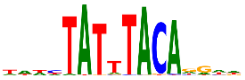	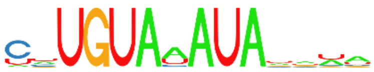
3'_modisco_07	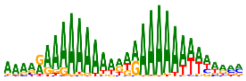	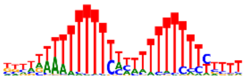	Repeat
3'_modisco_08	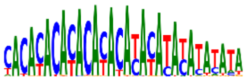	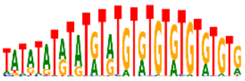	Repeat
3'_modisco_09, Kozak sequence	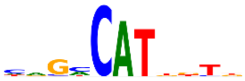	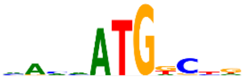	**aAaAaAATGTCt**
3'_modisco_10	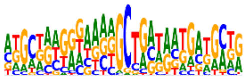	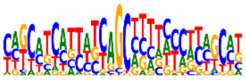	?
3'_modisco_11, Whi3 motif	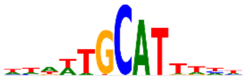	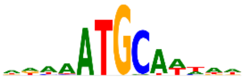	**UGCAU**
3'_modisco_12 SFP1	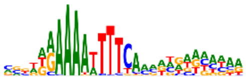	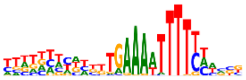	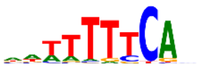
3'_modisco_13	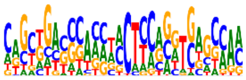	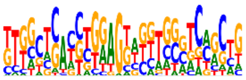	?
3'_modisco_14	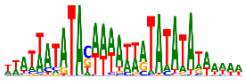	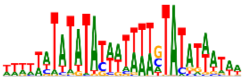	?
3'_modisco_15	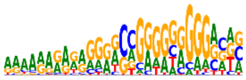	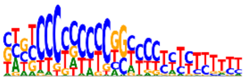	?
3'_modisco_16, E-box	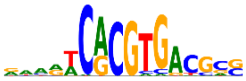	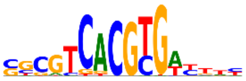	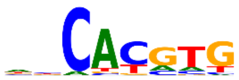
3'_modisco_17	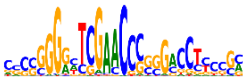	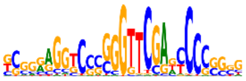	?

Name	Fwd	Rev	Match
3'_modisco_18	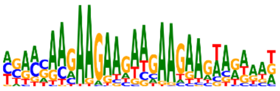	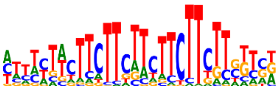	Repeat
3'_modisco_19	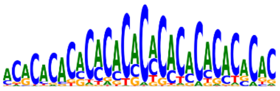	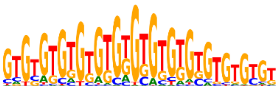	Repeat
3'_modisco_20	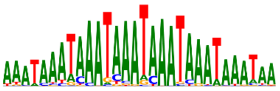	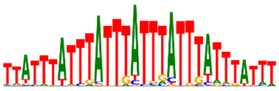	Repeat
3'_modisco_21	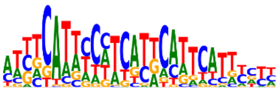	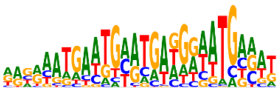	?
3'_modisco_22	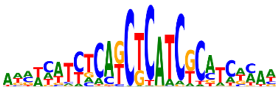	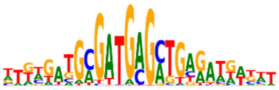	?
3'_modisco_23	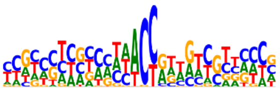	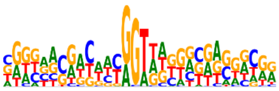	?
3'_modisco_24	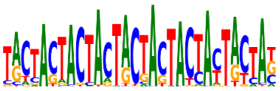	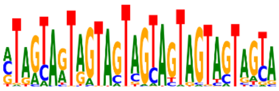	Repeat
3'_modisco_25	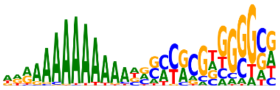	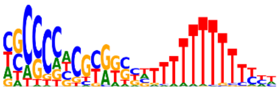	?
3'_modisco_26	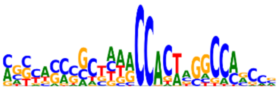	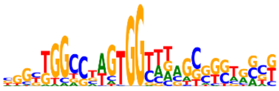	?
3'_modisco_27	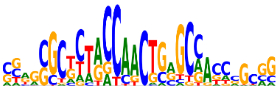	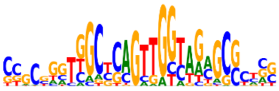	?
3'_modisco_28	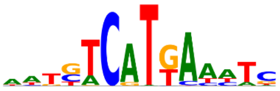	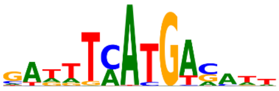	?
3'_modisco_29	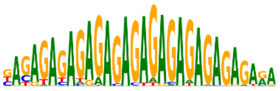	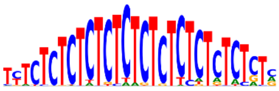	Repeat
3'_modisco_30	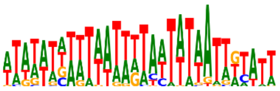	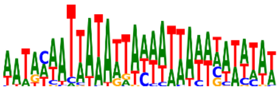	?

Modisco - 5'

Name	Fwd	Rev	Match
5'_modisco_00	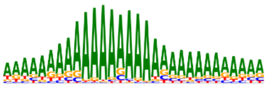	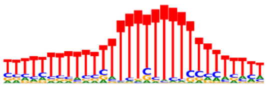	Repeat
5'_modisco_01, Kozak sequence	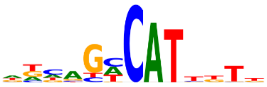	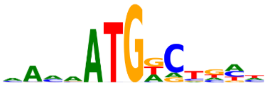	**aAaAaAATGTCt**
5'_modisco_02	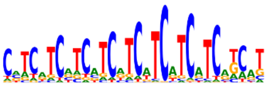	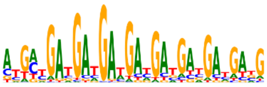	Repeat
5'_modisco_03	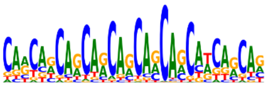	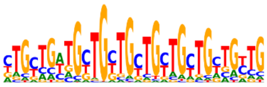	Repeat
5'_modisco_04	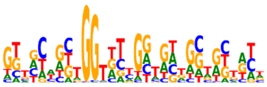	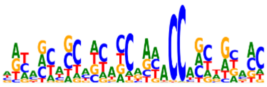	Repeat
5'_modisco_05, PAC (Tod6)	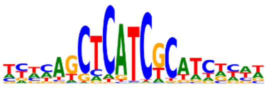	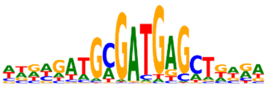	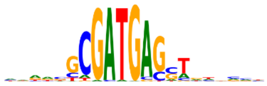
5'_modisco_06, Reb1	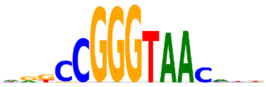	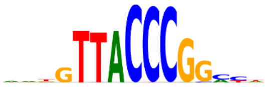	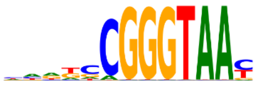
5'_modisco_07	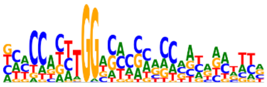	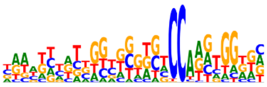	?
5'_modisco_08	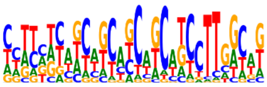	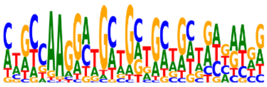	?
5'_modisco_09	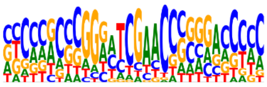	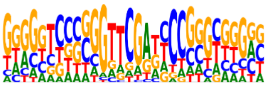	?
5'_modisco_10	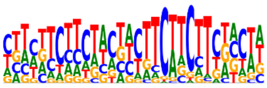	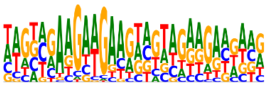	?
5'_modisco_11	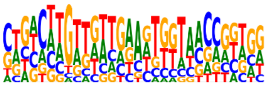	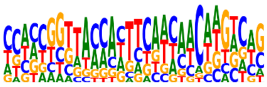	?
5'_modisco_12, TATA-box	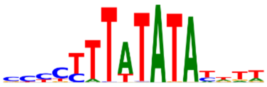	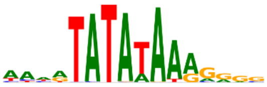	**TATAWAWR**

Name	Fwd	Rev	Match
5'_modisco_13	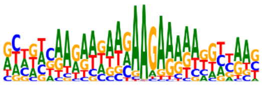	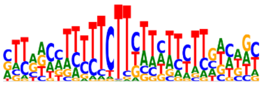	?
5'_modisco_14	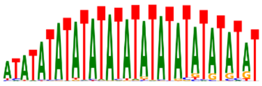	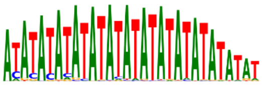	Repeat
5'_modisco_15	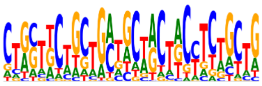	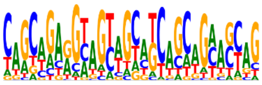	Repeat
5'_modisco_16, Rap1	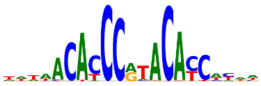	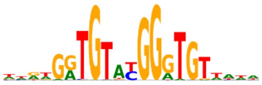	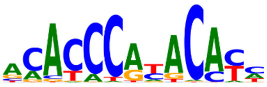
5'_modisco_17	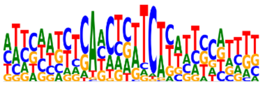	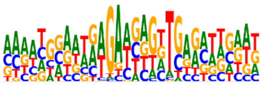	?
5'_modisco_18	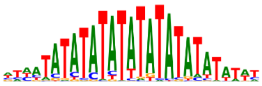	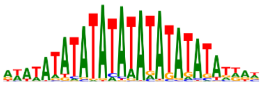	Repeat
5'_modisco_19	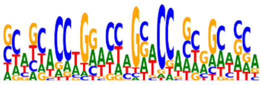	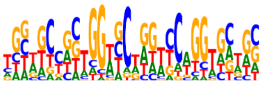	?
5'_modisco_20	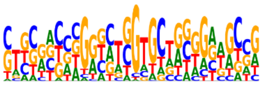	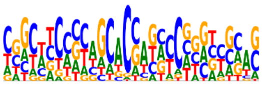	?
5'_modisco_21	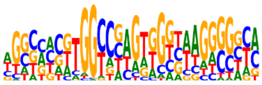	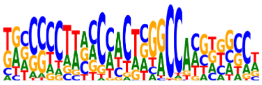	?
5'_modisco_22, SFP1	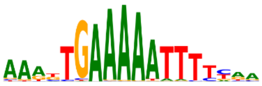	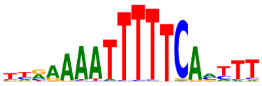	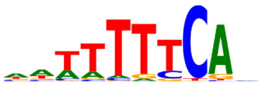
5'_modisco_23	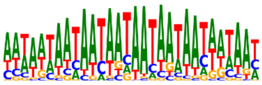	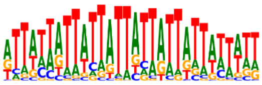	Repeat
5'_modisco_24, STB3	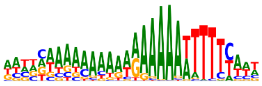	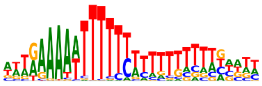	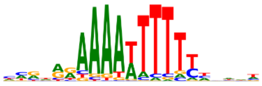
5'_modisco_25	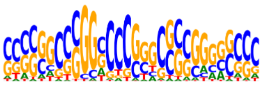	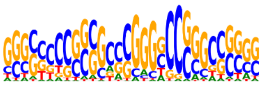	Repeat

Name	Fwd	Rev	Match
5'_modisco_26	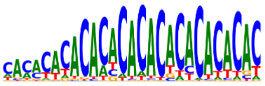	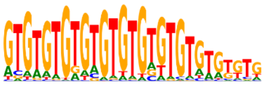	Repeat
5'_modisco_27, Reb1	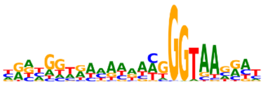	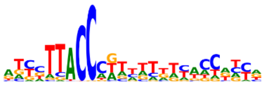	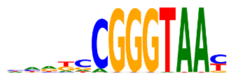
5'_modisco_28, Tbf1	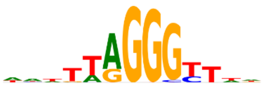	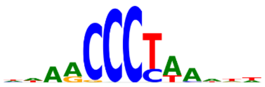	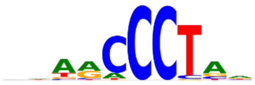
5'_modisco_29, E-box (Cbf1)	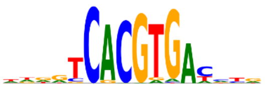	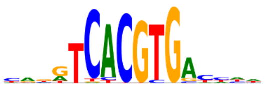	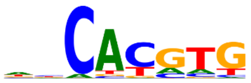
5'_modisco_30	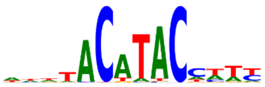	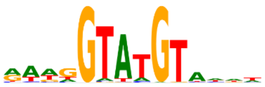	Splice donor
5'_modisco_31	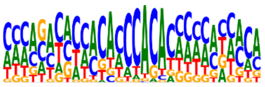	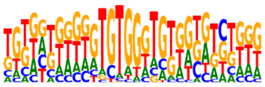	?
5'_modisco_32, Ume6	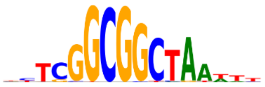	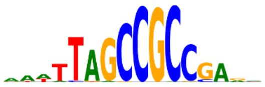	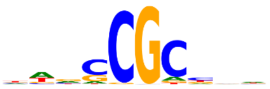
5'_modisco_33	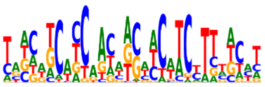	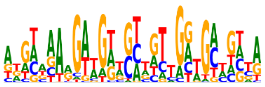	?
5'_modisco_34	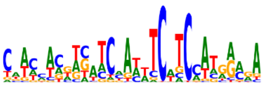	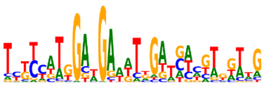	?
5'_modisco_35	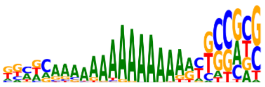	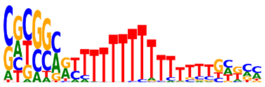	Repeat
5'_modisco_36, Mcm1	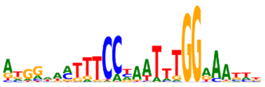	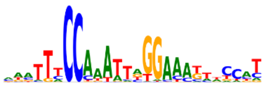	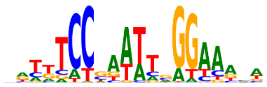
5'_modisco_37	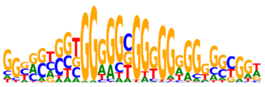	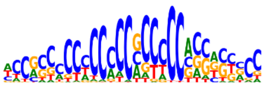	?
5'_modisco_38	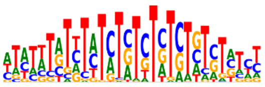	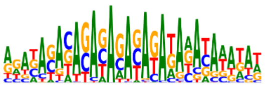	Repeat

Name	Fwd	Rev	Match
5'_modisco_39	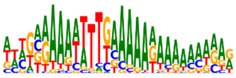	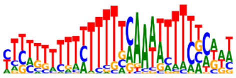	?
5'_modisco_40, U2	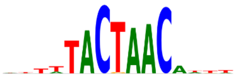	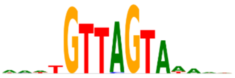	**Splice branchpoint**
5'_modisco_41	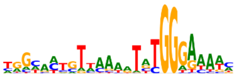	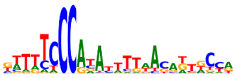	?
5'_modisco_41	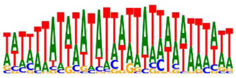	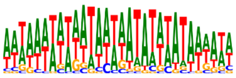	Repeat
5'_modisco_42, Mbp1	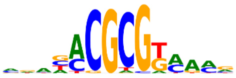	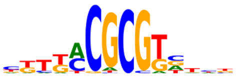	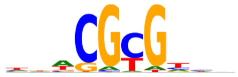
5'_modisco_43, Kozak sequence	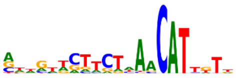	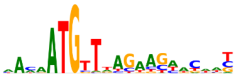	**aAaAaAATGTCt**
5'_modisco_44,	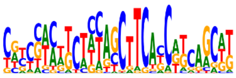	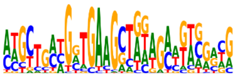	?
5'_modisco_45	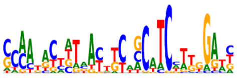	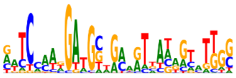	?
5'_modisco_46	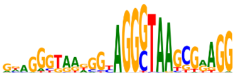	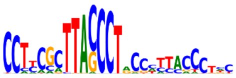	**?**
5'_modisco_47	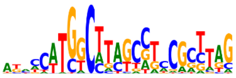	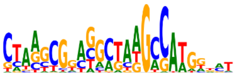	?
5'_modisco_48, Fkh1/2	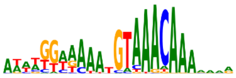	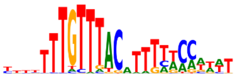	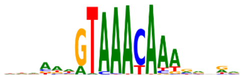
5'_modisco_49, Puf3	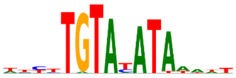	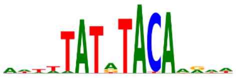	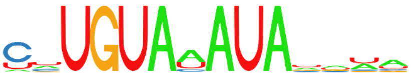
5'_modisco_50	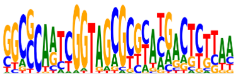	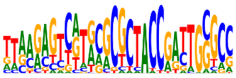	?

Streme - 3’

Name	Fwd	Match
3'_streme_00	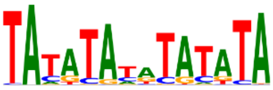	Repeat
3'_streme_01	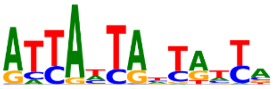	?
3'_streme_02	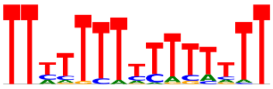	Repeat
3'_streme_03	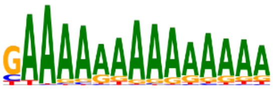	Repeat
3'_streme_04	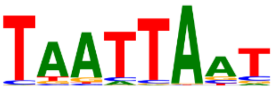	?
3'_streme_05	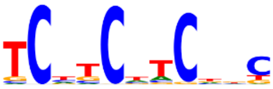	Repeat
3'_streme_06, Reb1	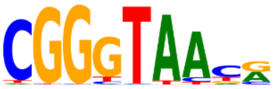	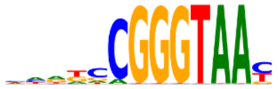
3'_streme_07	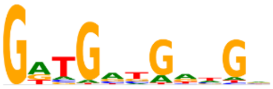	Repeat
3'_streme_08, PAC (Tod6)	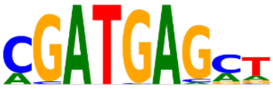	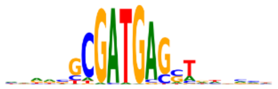
3'_streme_09	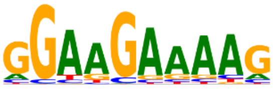	?
3'_streme_10, Pin4	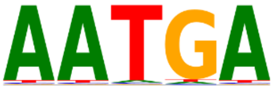	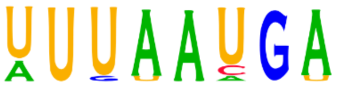
3'_streme_11	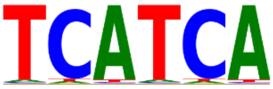	?
3'_streme_12	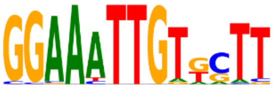	?
3'_streme_13	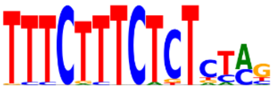	?
3'_streme_14	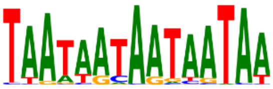	?

Streme - 5’

Name	Fwd	Rev	Match
5’_streme_00	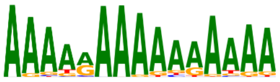	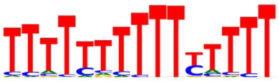	Repeat
5’_streme_01	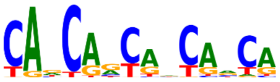	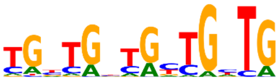	Repeat
5’_streme_02	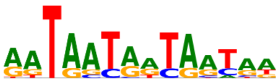	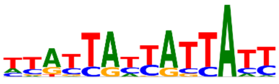	Repeat
5’_streme_03	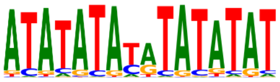	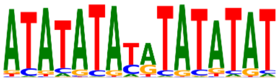	Repeat
5’_streme_04	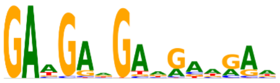	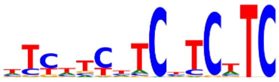	Repeat
5’_streme_05	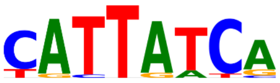	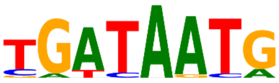	?
5’_streme_06, Reb1	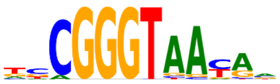	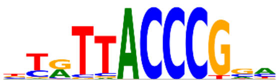	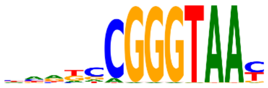
5’_streme_07	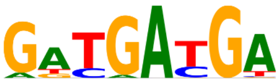	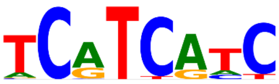	?
5’_streme_08, Ume6	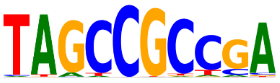	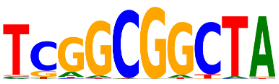	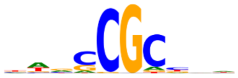
5’_streme_09, Stb3	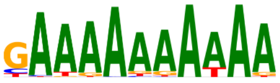	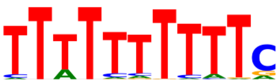	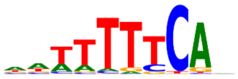
5’_streme_10	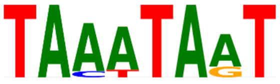	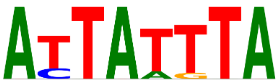	?
5’_streme_11	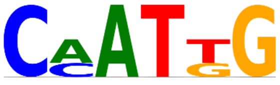	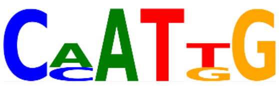	?
5’_streme_12, Mcm1	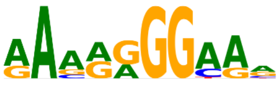	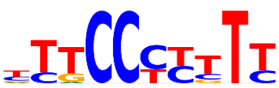	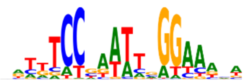
5’_streme_13	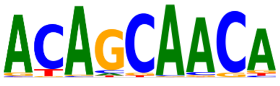	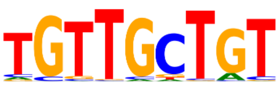	?

Name	Fwd	Rev	Match
5'_streme_14	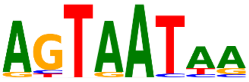	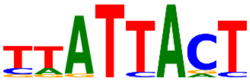	?
5'_streme_15	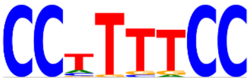	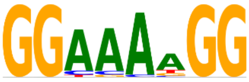	?
5'_streme_16, Dot6	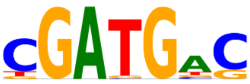	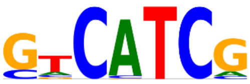	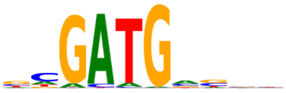
5'_streme_17	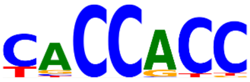	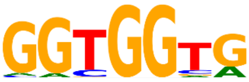	?
5'_streme_18	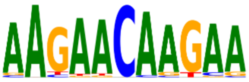	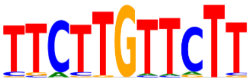	?
5'_streme_19	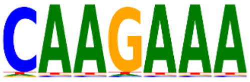	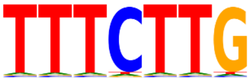	?
5'_streme_20, Tod6	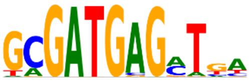	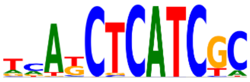	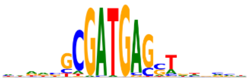
5'_streme_21	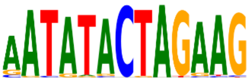	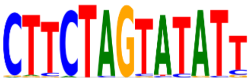	?
5'_streme_22	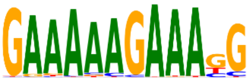	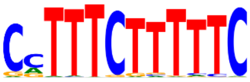	?
5'_streme_23	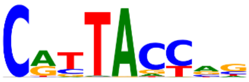	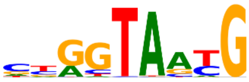	?
5'_streme_24, Stb3	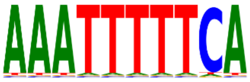	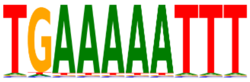	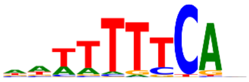
5'_streme_25	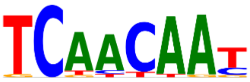	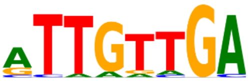	?
5'_streme_26, Xbp1	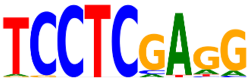	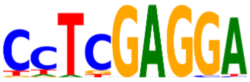	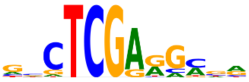
5'_streme_27	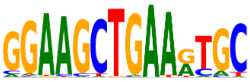	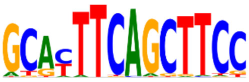	?

Name	Fwd	Rev	Match
5'_streme_28	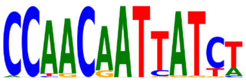	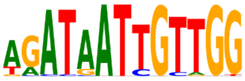	?
5'_streme_29	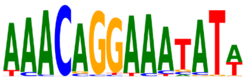	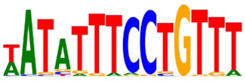	?
5'_streme_30	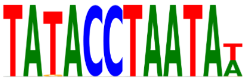	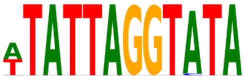	?
5'_streme_31	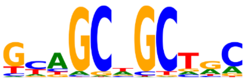	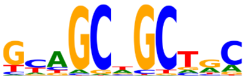	?
5'_streme_32	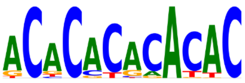	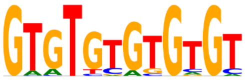	?
5'_streme_33	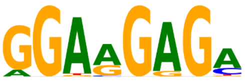	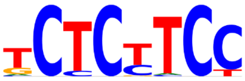	?
5'_streme_34	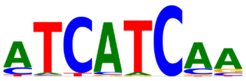	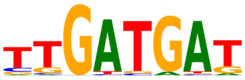	?
5'_streme_35	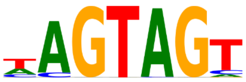	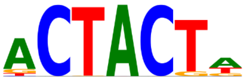	?
5'_streme_36, Rap1	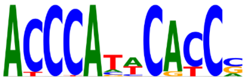	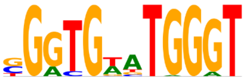	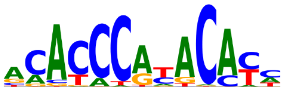
5'_streme_37	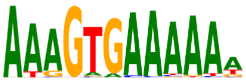	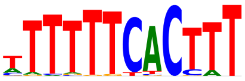	?
5'_streme_38	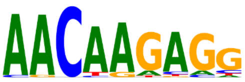	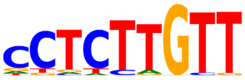	?

Species Token Choice

During model training, we hold out the Saccharomyces genus, so as to measure the ability of the DNA LM to generalize to unseen species. However, the species LM requires a species token to make predictions, so we must supply it with a proxy token for the held-out species. This document explores the impact of different choices for the proxy token.

Intuitively, we expect that species which are phylogenetically closer to the held-out species should provide better proxy tokens, since they likely share more features of the regulatory code. Choosing proxies in this way is not circular, since the phylogeny is not provided to the model and moreover these phylogenies are generally computed using protein sequences, rather than flanking regions. Fig S14 shows the phylogenetically closest species to S. *cerevisiae* in our training set. These include *C. glabrata*, the genera Kazachstania and Naumovozyma as well as the genera Vanderwaltozyma and Tetrapisispora - which together comprise the set of post-whole-genome-duplication yeasts in our training dataset.

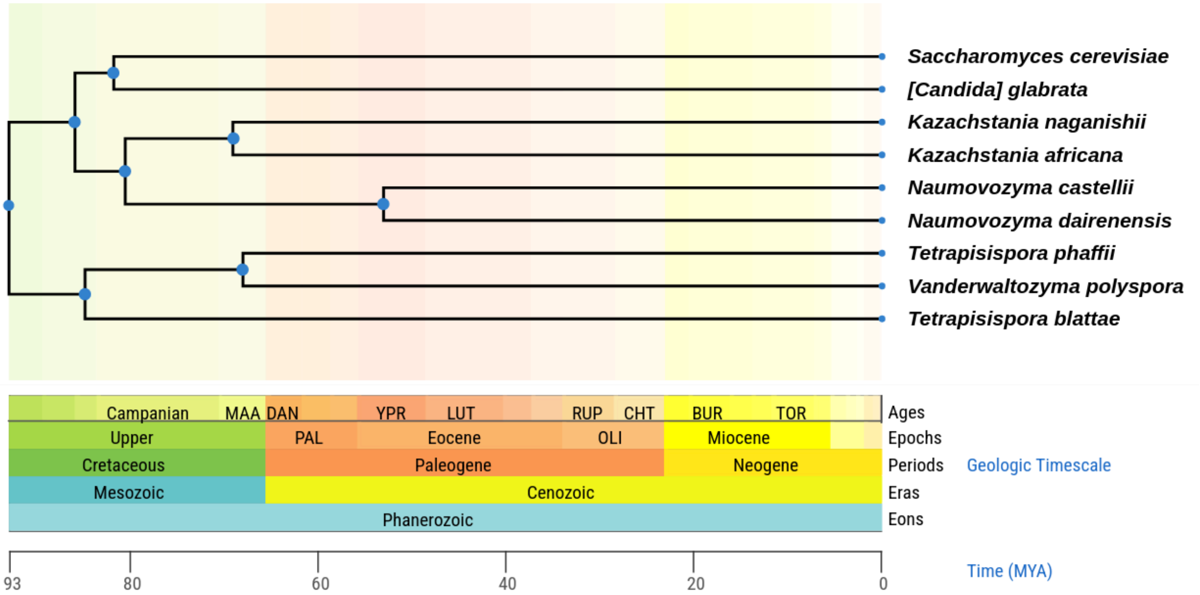

Fig S14: Phylogenetic tree depicting our held-out species S. *cerevisiae* and the species in our training dataset which are phylogenetically closest to S. *cerevisiae*. Figure created using http://www.timetree.org/

For the most part, any of these species can be used as a proxy for *S. cerevisiae*. Performance on motif reconstruction (Fig S15, S16) and predictive tasks (Table S3) will of course vary depending on the token used, however, even if we always take the worst token from these species for each motif or predictive task, the species LM will still generally perform well. Accordingly, the main conclusions of the paper are robust to any reasonable choice of species proxy token.

We note also that using highly diverged species as proxy, e.g. molds (Aspergillus or Penicillium) or Basidiomycota (Agaricus or Amanita), performs generally very poorly (Fig S17, Table S3).

As *C. glabrata* is phylogenetically closest to *S. cerevisiae*, it would appear to be the natural choice for the proxy token. However, this proxy performs uncharacteristically poorly for the 5' regions, particularly the E-box motif where it is a clear outlier compared to all other post-WGD species (Fig S16). Looking at the predictions made by the species LM for S. *cerevisiae* if *C. glabrata* is used as proxy, we found that the predictions show a skew towards CAC**A**TG, rather than CAC**G**TG. Interestingly, this appears to reflect the actual occurrence of these motifs in the C. *glabrata*

genome. Specifically, compared to a null model of shuffled sequences, CAC**G**TG is notably less enriched in C. *glabrata* than in the other post-WGD yeast, whereas CAC**A**TG is slightly more enriched than in the other post-WGD yeasts (Fig S18). In other words, the poor performance on the E-box of the species LM when the *C. glabrata* token is used is not a failure of the species LM, but rather likely a consequence of the species token correctly reflecting the species it is supposed to represent. To avoid such species idiosyncrasies when presenting results, we used the overall reconstruction accuracy on *S. cerevisiae* as a guide to select the proxy from among the phylogenetically close species. Through this procedure, *K. africana* was selected as proxy of choice for the 5' species LM.

As an alternative to selecting a single close species from the training set for prediction on an unseen species, a simple method is to average model predictions over several phylogenetically close species, e.g. the closest five (*C. glabrata, K. Africana, K. Naganishii, N. Castellii and N. dairenensis*) or all post-WGD species. The idea is that this averaging allows the model to represent the general features of this part of the phylogeny, without being too skewed towards the specifics of a particular species. This results in very good performance on nucleotide reconstructions (Fig S19, S20), TF-binding site classification (Fig S21) and predictive tasks (Table S3).

Another alternative method, which sidesteps proxy selection entirely, is to train an additional species token for *S. cerevisiae* - while leaving the remaining parameters of the species LM unchanged - but only on a part of the S. cerevisiae genome - in our case Chromosomes I-VII. One can then evaluate on the remainder of the *S. cerevisiae* genome (after removal of within-genome paralogues of the training genes). Since only 768 parameters need to be fitted, this token finetuning can be done with minimal computational cost. The resulting token allows the species LM to achieve decent reconstruction on most motifs (Fig S22, S23). We note, however, that the resulting token is likely somewhat undertrained as it has seen less than half of a genome and as a result some subset of motifs may not be encountered in sufficient quantity.

Altogether these investigations showed that averaging predictions using a set of close species is the most robust approach. However, all three approaches (single closest species, averaging across close species and learning a token on half of the genome) perform similarly well.

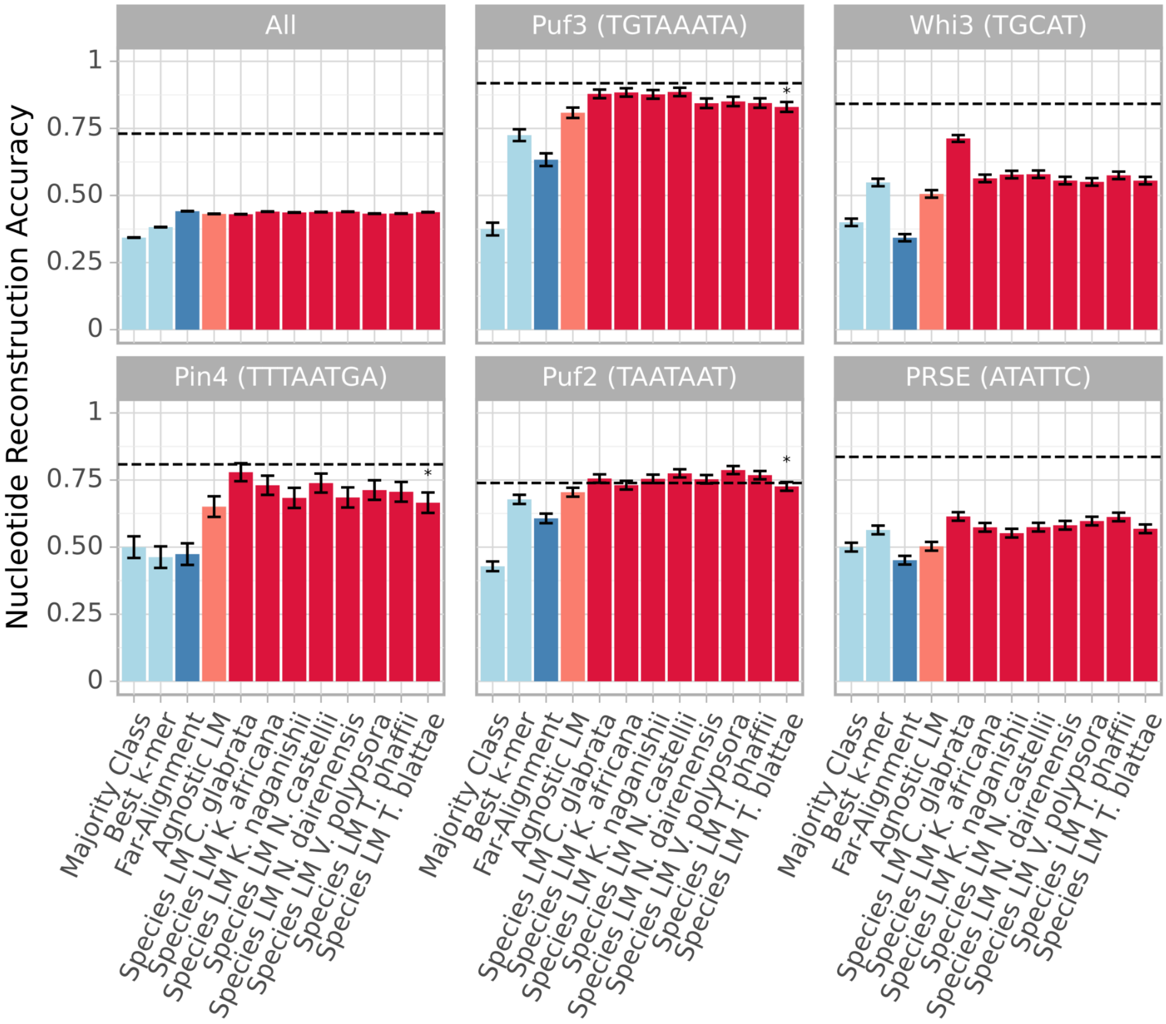

Fig S15: If phylogenetically close species are used as proxy, the species LM reconstruct likely regulatory sequences in the 3' regions of S. *cerevisiae*. Reconstruction accuracy for nucleotides within instances of RNA-binding protein consensus motifs and across all nucleotides in *S. cerevisiae* 3' UTR sequences (those longer than 300 bp have been truncated). We compare a variety of baselines, the agnostic 3' LM and species 3' LM with different proxy tokens corresponding to species which are phylogenetically close to S. *cerevisiae*. Dashed line represents the accuracy achieved by the intra-genus alignment. Star indicates that the species LM with the *worst* performing proxy-token significantly ($P < 0.05$, binomial test) outperforms the *best* non-LM baseline. We observe that using C. *glabrata*, which is closest to S. *cerevisiae*, as a proxy species generally performs best on the various motifs. However, even if we take the worst proxy, the species LM nevertheless significantly outperforms the best non-LM baseline on 3 out of 5 motifs.

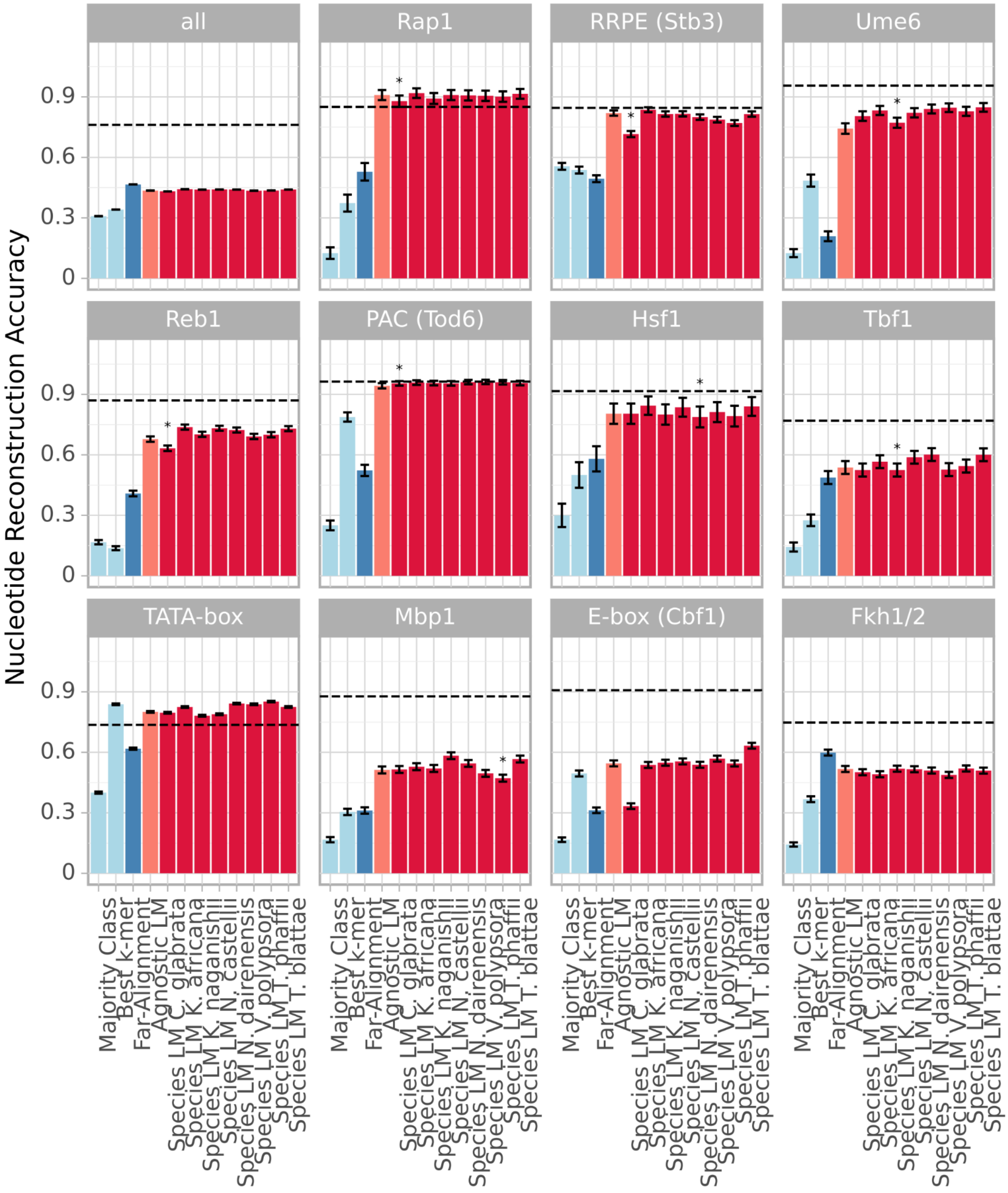

Fig S16: If phylogenetically close species are used as proxy, the species LM reconstruct likely regulatory sequences in the 5' regions of S. *cerevisiae*. Reconstruction accuracy for nucleotides within instances of Transcription Factor consensus motifs and across all nucleotides in *S. cerevisiae* 5' regions. We compare a variety of baselines, the agnostic 5' LM and species 5' LM with different proxy tokens corresponding to species which are phylogenetically close to S. *cerevisiae*. Dashed line represents the accuracy achieved by the intra-genus alignment. Star indicates that the species LM with the *worst* performing proxy-token significantly (P < 0.05, binomial test) outperforms the *best* non-LM baseline. We observe that performance varies somewhat across species and particularly using C. *glabrata* as proxy-species tends to perform very poorly for the E-box. However, even if we use the worst proxy each time, the species LM still decisively beats the non-LM baselines in 8/11 motifs shown.

	Proxy species for Species LM	*S. cerevisiae* RNA half-life Cheng et al.	*S. cerevisiae* MPRA Shalem et al.	*S. cerevisiae* gene expression, Zrimec et al. 3’	*S. cerevisiae* gene expression, Zrimec et al. 5’	*S. cerevisiae* gene expression, Zrimec et al. 3’ + 5’	Promoter MPRA, Keren et. al
Far species	*A. bisporus*	0.572	0.441	0.166	0.198	0.288	0.191
	A. muscaria	0.574	0.471	0.237	0.283	0.386	0.247
	A. niger	0.576	0.512	0.182	0.211	0.311	0.273
	A. fumigatus	0.575	0.501	0.181	0.204	0.314	0.326
	N. crassa	0.573	0.491	0.210	0.205	0.335	0.303
Post-WGD species	*V. polyspora*	**0.594**	**0.687**	**0.300**	**0.462**	**0.538**	0.415
	T. blattae	**0.590**	**0.671**	**0.299**	**0.466**	**0.528**	0.436
	T. phaffii	**0.594**	**0.690**	**0.297**	**0.444**	**0.525**	0.414
	N. dairenensis	**0.59**	**0.694**	**0.295**	**0.454**	**0.522**	0.430
	N. castellii	**0.591**	**0.687**	**0.302**	**0.472**	**0.536**	**0.447**
	K. naganishii	**0.588**	**0.676**	**0.288**	**0.44**	**0.517**	0.427
	K. africana	**0.592**	**0.695**	**0.299**	**0.492**	**0.551**	**0.449**
	C. glabrata	**0.592**	**0.704**	**0.297**	**0.455**	**0.532**	0.429
Ensemble	Post-WGD species	**0.600**	**0.716**	**0.309**	**0.481**	**0.551**	**0.442**
	Five closest species	**0.599**	**0.721**	**0.306**	**0.480**	**0.549**	**0.449**

Table S3: Species LM performance on *S. cerevisiae* predictive tasks for a variety of different proxy species. Bold indicates that the model outperforms both the relevant SOTA and the agnostic LM, whereas underline indicates the best overall. For all but one task, any post-WGD species can be used as a proxy and the species LM will outperform all baselines. By contrast, if phylogenetically far-species are used as proxy, the species LM performs very poorly. If we average predictions across the five phylogenetically closest species or across all post-WGD species, the species LM outperforms both the agnostic LM and the SOTA on all tasks.

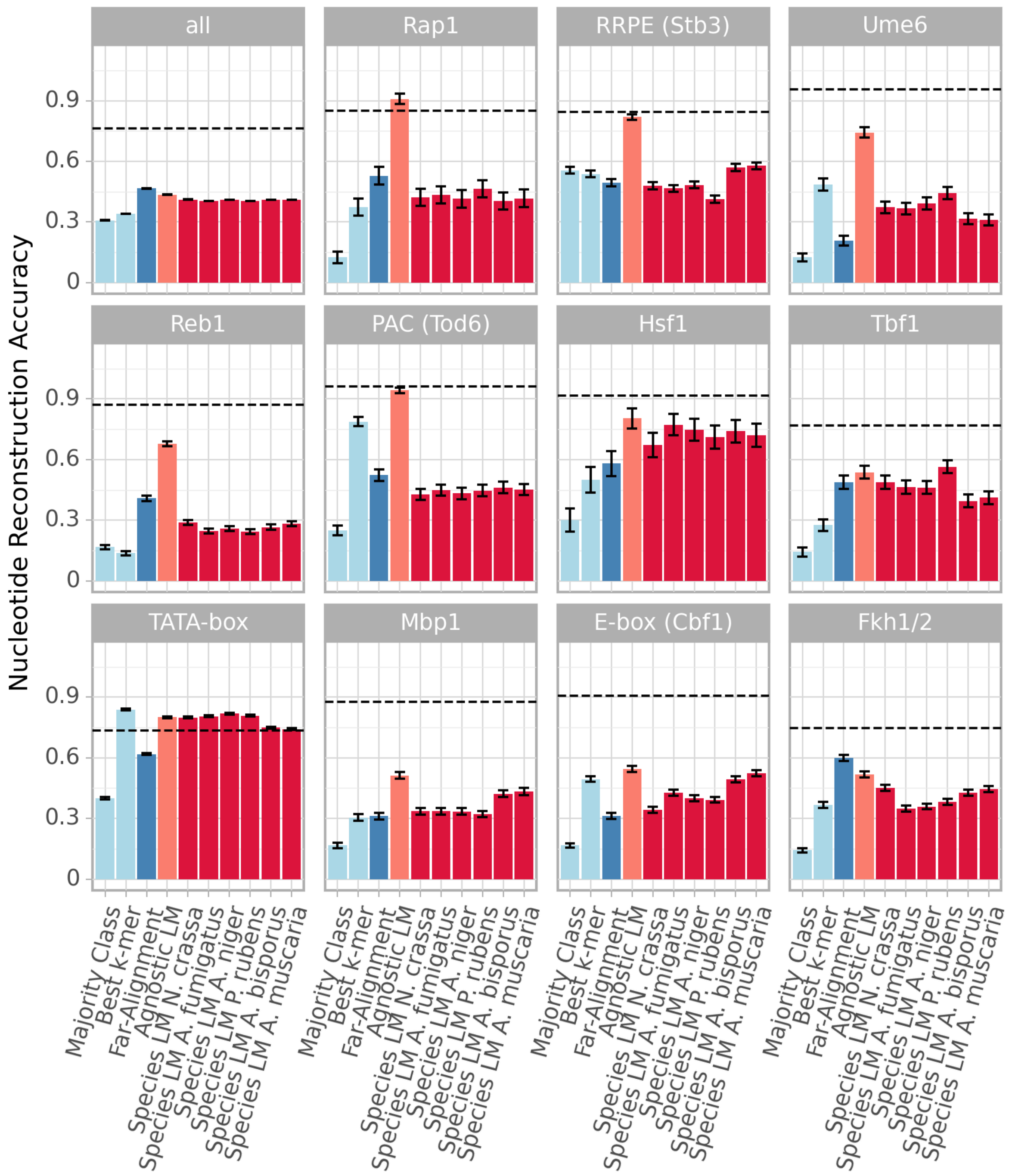

Fig S17: If phylogenetically far species are used as proxy, the species LM reconstructs poorly. Reconstruction accuracy for nucleotides within instances of Transcription Factor consensus motifs and across all nucleotides in *S. cerevisiae* 5' regions. We compare a variety of baselines, the agnostic 3' LM and species 3' LM with different proxy tokens corresponding to species which are phylogenetically and phenotypically far from S. *cerevisiae*. These species include molds such as Aspergillus and Penicillium, as well as two Basidiomycota. For most motifs, using these far species as a proxy leads to very poor reconstructions.

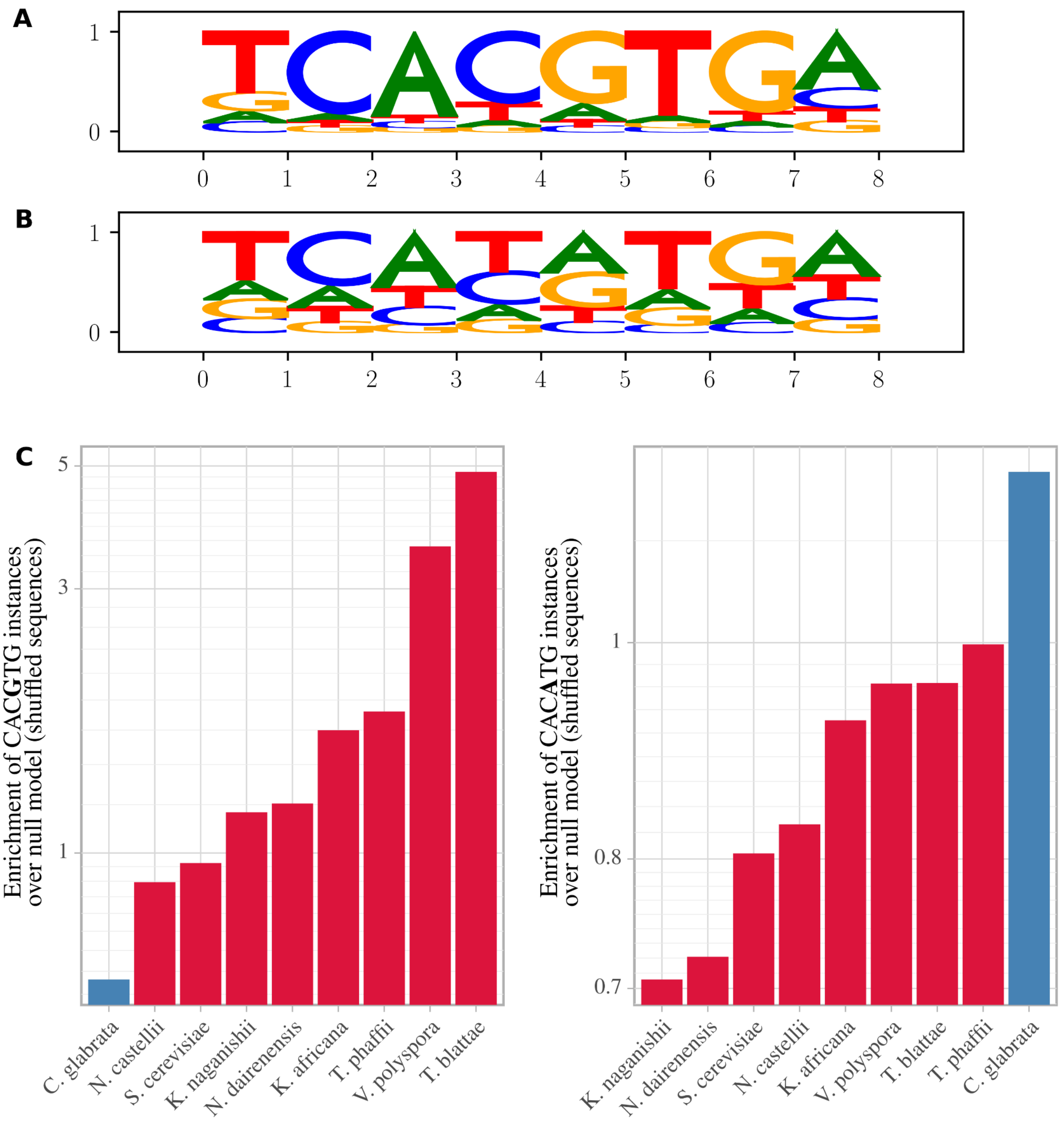

Fig S18: Poor reconstruction of the Ebox when *C. glabrata* is used as proxy token may reflect relative lack of enrichment of this motif in *C. glabrata*. A) Predicted probabilities provided by the species LM for nucleotides in binding E-box motifs in S. cerevisiae. Here *K. africana* is used as proxy species. Note that to predict, we always mask one nucleotide. Thus the height of the letters represents the predicted probability conditional on the model seeing the correct nucleotides at all other positions **B)** Same as A, but *C. glabrata* is used as proxy species. We observe a preference for CAC**A**TG (reverse complement CA**T**GTG). **C)** Enrichment of CAC**G**TG and CAC**A**TG vs. a null model of shuffled sequences in a variety of post-WGD species. We observe that in *C. glabrata*, there is a notably lower enrichment of CAC**G**TG and a higher enrichment of CAC**A**TG. Accordingly, the tendency of the model to predict CAC**A**TG/CA**T**GTG instead of CAC**G**TG when *C. glabrata* is used as proxy appears to reflect the k-mer distribution within that species.

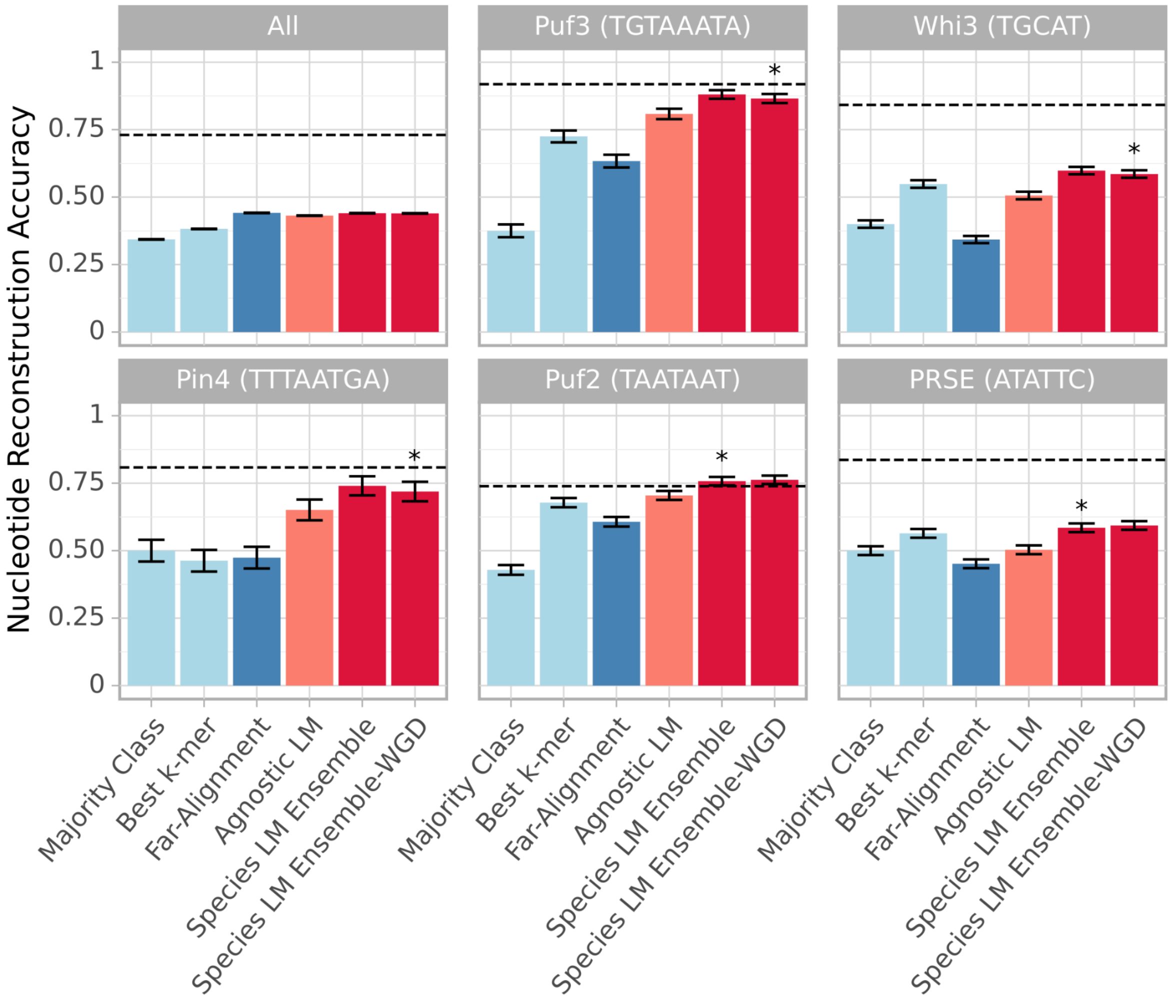

Fig S19: If we average the reconstructions for several close species, the species LM performs well on all 3' motifs. Reconstruction accuracy for nucleotides within instances of Transcription Factor consensus motifs and across all nucleotides in *S. cerevisiae* 3' UTR sequences (those longer than 300 bp have been truncated). We compare a variety of baselines, the agnostic 3' LM and the species 3' LM, where we average across the reconstructions made with several close species - either the 5 closest (Species LM Ensemble) or all post-WGD species (Species LM Ensemble-WGD). Dashed line represents the accuracy achieved by the intra-genus alignment. Star indicates that the *worst* performing species LM significantly ($P < 0.05$, binomial test) outperforms the *best* non-LM baseline. This is the case for all motifs.

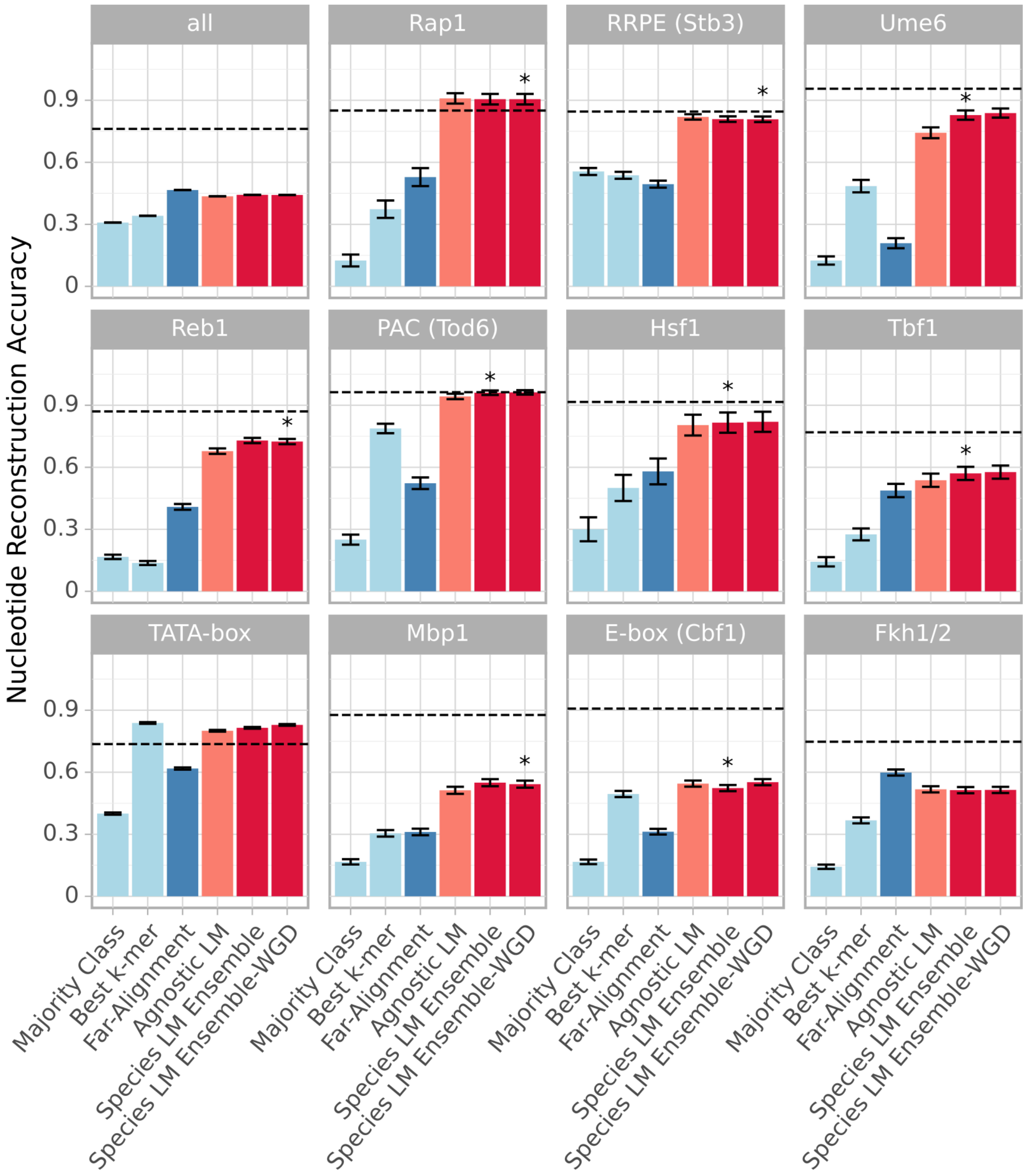

Fig S20: If we average the reconstructions for several close species, the species LM performs well on 5' motifs. Reconstruction accuracy for nucleotides within instances of Transcription Factor consensus motifs and across all nucleotides in *S. cerevisiae* 5' regions. We compare a variety of baselines, the agnostic 5' LM and the species 5' LM, where we average across the reconstructions made with several close species - either the 5 closest (Species LM Ensemble) or all post-WGD species (Species LM Ensemble-WGD). Dashed line represents the accuracy achieved by the intra-genus alignment. Star indicates that the *worst* performing species LM significantly (P < 0.05, binomial test) outperforms the *best* non-LM baseline. This is the case for 9 out of 11 motifs.

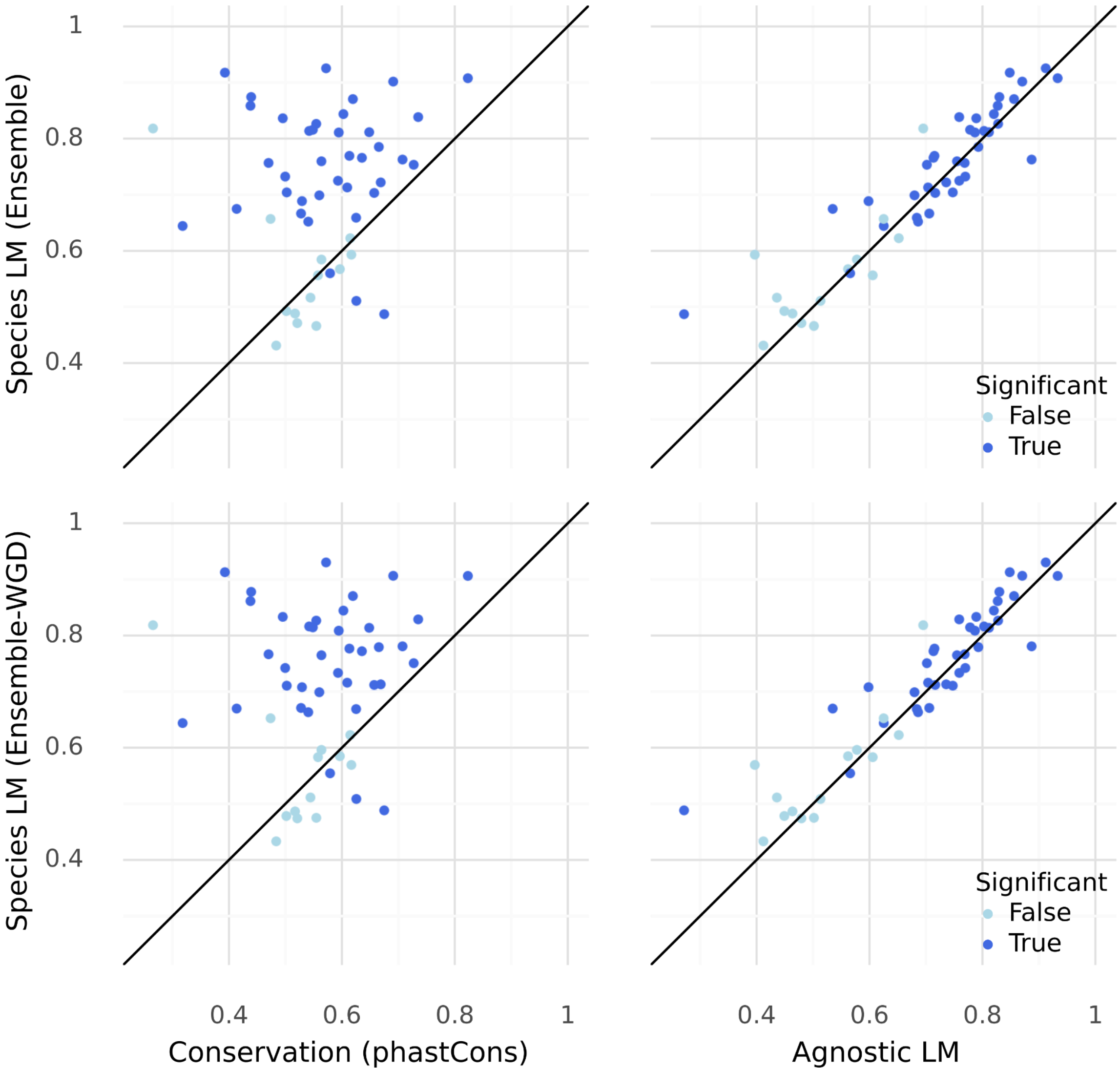

Fig S21: The predictiveness of the species LM reconstructions for in-vivo binding generalizes across a variety of yeast TF. We evaluated to what extent the reconstruction fidelity of our 5' LMs, as well as the phastCons conservation score, can serve as a predictor of whether consensus motif instances of a variety of TF are bound in-vivo. For this we analyzed all yeast TF which (1) show more than 100 peaks in the Rossi et al. CHIP-exo dataset and (2) have a motif of at least medium confidence in the YeTFaSCo database. Altogether these are 51 TF. In the upper left panel, we compare the AUROC achieved per TF of the species LM (with the reconstructions averaged across the closest five species) with that achieved by the conservation score. Each point represents one TF. Dark blue color indicates that the AUROC achieved by at least one of the methods being compared is significantly different from random (P < 0.05, Mann-Whitney U). Altogether, the species LM decisively outperforms the phastCons score ($P = 9.5*10^{-7}$, paired WIlcoxon). The upper right panel compares against the agnostic LM, which again performs slightly worse than the species LM (P = 0.024). The lower two panels compare the species LM (with reconstructions averaged across all post-WGD species) against the phastCons score ($P = 8.2*10^{-7}$) and the agnostic LM (P = 0.008).

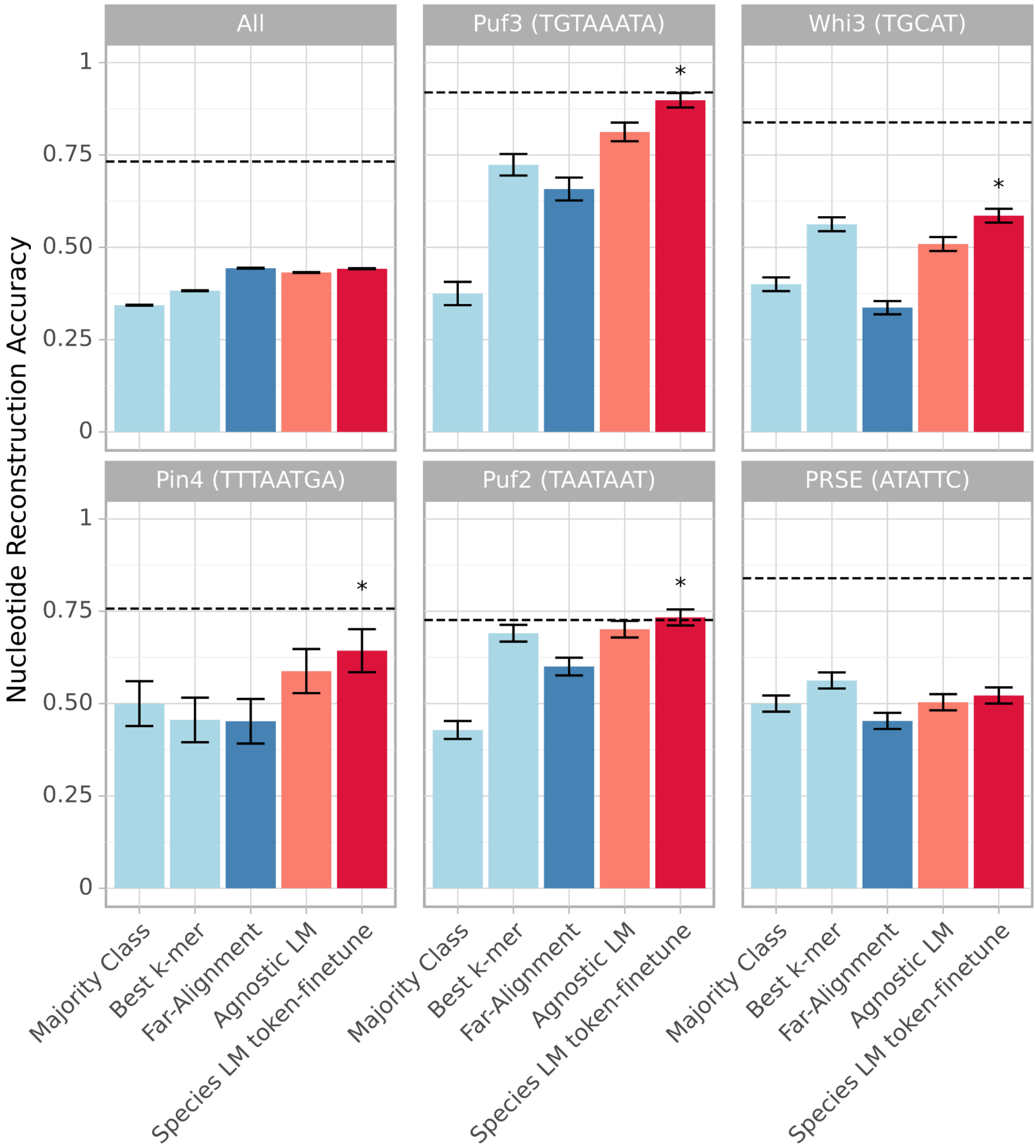

Fig S22: Training a *S. cerevisiae* token on Chromosomes I-VII enables the species LM to reconstruct well on 3' motifs of the remaining chromosomes. Reconstruction accuracy for nucleotides within instances of Transcription Factor consensus motifs and across all nucleotides in *S. cerevisiae* 3' UTR sequences (those longer than 300 bp have been truncated) of genes in Chromomes VII-XVI. We compare a variety of baselines, the agnostic 3' LM and the species 3' LM, where we use a species token trained on Chromosomes I-VII. Dashed line represents the accuracy achieved by the intra-genus alignment. Star indicates that the species LM significantly ($P < 0.05$, binomial test) outperforms the *best* non-LM baseline. This is the case for 4 out of 5 motifs.

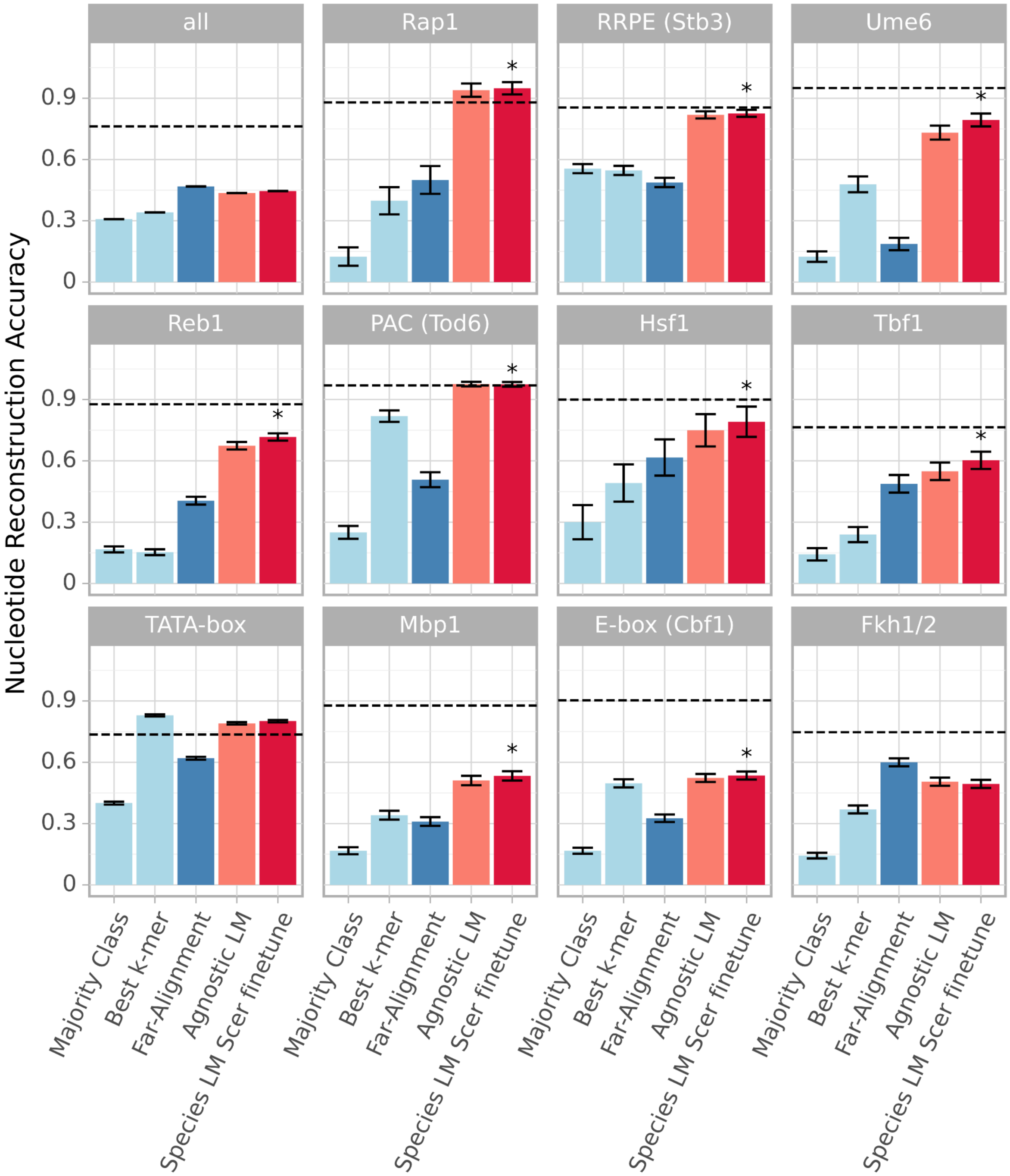

Fig S23: Training a *S. cerevisiae* token on Chromosomes I-VII enables the species LM to reconstruct well on 5' motifs of the remaining chromosomes. Reconstruction accuracy for nucleotides within instances of Transcription Factor consensus motifs and across all nucleotides in *S. cerevisiae* 5' regions of genes in Chromomes VII-XVI. We compare a variety of baselines, the agnostic 5' LM and the species 5' LM, where we use a species token trained on Chromosomes I-VII. Dashed line represents the accuracy achieved by the intra-genus alignment. Star indicates that the species LM significantly ($P < 0.05$, binomial test) outperforms the *best* non-LM baseline. This is the case for 9 out of 11 motifs.
