# Supplementary material for: Species-aware DNA language models capture regulatory elements and their evolution
Source: Genome Biol. 2024 Apr 2;25:83. doi: 10.1186/s13059-024-03221-x (PMC10985990; doi:10.1186/s13059-024-03221-x)
Supplement: Supplementary file 3 — Additional file 3. Peer review history. [file 13059_2024_3221_MOESM3_ESM.docx]

**Review history**

**First round of review**

**Reviewer 1**

Karollus, et al. present an application of large language models (LLMs) to learn features of DNA sequences in non-coding regions across hundreds of yeast genomes. They then investigate how well the LLMs can recover masked sequence examples, particularly focusing on the S. cerevisiae genome that was unseen during training. Remarkably, despite having not been provided any form of regulatory information during training, the LLMs perform better at reconstructing functional regulatory elements such as TF or RBP binding sites. The manuscript thus claims that the LLMs are "capturing" regulatory information within the genome sequences.
Several other publications have applied LLMs to non-coding sequences over the past few years, and the current manuscript does not claim to advance the underlying neural network methodology (it uses the DNABERT LLM model). Rather, the innovations in the current manuscript include how the LLMs have been applied across a large collection of genome sequences. And in particular, this manuscript proposes LLM reconstruction accuracy as a metric for assessing regulatory conservation and innovation across species.
Overall, I think this manuscript presents a powerful application of LLMs to uncover regulatory signals across species. I believe it will be of great interest to the readership of Genome Biology. I have relatively few comments about the manuscript.

1) The results presented in the manuscript are impressive; reconstruction accuracy scores passed into TF-MoDISco enables the detection of TF and RBP binding motifs in S. cerevisiae regulatory regions, and the models appear to learn spatial contexts of some motifs (e.g., higher reconstruction accuracy of the TATA box near TSSs). However, it is difficult to get a sense for how generalizable these results are, or whether we are being presented the cherry-picked best cases. Fortunately, an almost-complete set of S. cerevisiae TF binding sites have been characterized in high resolution under YPD conditions (Rossi, et al., Nature 2021). While the current manuscript uses some of these ChIP-exo datasets in evaluations, there would appear to be an opportunity to more globally assess sensitivity and specificity of reconstruction accuracy compared with other metrics such as phyloP. Obviously, a caveat would apply to specificity calculations, since non-YPD condition-specific TF binding sites could exist that have not been profiled by Rossi, et al.

2) Providing the species label during training enables the LLMs to more accurately capture species-specific regulatory signals. What do the species labels enable the models to learn? I presume the models learn to vary the oligonucleotide composition changes based on the species label provided, but could this be demonstrated? For example, by examining how "reconstructions" of random sequences vary dependent on the species label.

3) Could the authors comment on whether adding information about gene homology could also help the LLMs to learn more specific regulatory features? For example, if genes were grouped by homology, could per-group labels be provided during training?

**Reviewer 2**

In this manuscript, the authors explore the utility of DNA language models (specifically, the DNABERT implementation from Ref. 14) for annotating non-coding genomic sequence in terms of where cis-regulatory elements are expected to occur relative to genomic landmarks such as a gene's transcriptional start site or UTRs. According to the authors, it is not yet computationally feasible to do this for mammalian genomes, so they do this for a set of 800 fungal genomes.

This is an interesting and useful study that gives authors a concrete practical sense of what a LM can offer. Predictive performance takes the form of the likelihood of correctly reconstructing the true base identity at masked genomic positions based on knowledge of the actual local genomic sequence context. Since the surrounding sequence contains features that are correlated with spatial landmarks, it is perhaps not surprising that overall, predicted spatial patterns (such as in Figure 3A) are consistent with expectation. The most interesting analysis in my view were those where functionality of regulatory elements was predicted from local sequence context, e.g., whether a given consensus sequence was bound by a TF or not (Figure 3D), or the use of the LM embedding as a predictor in regression mode (Figure 5).

I have one major technical concern that needs to be addressed by the authors in a convincing manner: The popular model yeast Saccharomyes cerevisiae, for which a plethora of functional data is available, plays a special role, in that it is held out from the training set of the LM, so that it can serve as a test set. Now, on page 16 the authors state that in order to use their species-aware LM to make predictions of regulatory for the species-aware version of their model to S. cerevisiae, they had to select a proxy species "based on overall reconstruction achieved." This sounds to me like a basic violation of the need for strict separation between testing and training set. This is particularly relevant since the authors repeatedly comment that the performance of the species-aware LM relative to the species-agnostic one is "remarkable." Maybe it is remarkable for the wrong reasons, especially when a best-performing proxy species can be chosen from among 800 candidates?
Note that a possible solution to this problem would be to not hold out one entire genome, but to hold out a large number (say, 50%) of S. cerevisiae genes from the training set, while keeping the other 50% as part of the training set, so that the species-specific features can be learned for S. cerevisiae, and the problematic selection of a proxy species is avoided altogether.

Further comments:

- Page 3, "after fine-tuning": Can you give some intuition what this means in practice? In general, it would be great if the authors would not assume that the readers are already intimately familiar with language models, and provide some more detailed explanation and intuition of how these models work. The diagrams in Figure 1C and 1D are not as informative as they could be perhaps.
- Page 5, "the need and potential for alignment-free approaches": Alignment-free approaches have been used in the past to analyze non-coding genomic DNA. See for instance PMID 17993685, 16809671, 18586710. It may be useful to cite some of that older work here. There are also older methods akin to language models that have been applied to find regulatory elements based on a statistical analysis non-coding sequence alone (e.g., PMID 10944202) that the readers of this manuscript may find interesting.
- When building the LM, were all genes analyzed jointly, or was the LM build separately for all orthologs of the regulatory region of each individual gene? It is not 100% clear from the text, so define this more explicitly. Could it be useful for build per-gene LM's?
- Page 7, "we were able to recover some of these motifs de novo": So are these cherry-picked examples? To counter this impression, it would be good if the success rate across all known consensus motifs could be reported here, and how this rate compares to that of a reference "standard" approach.
- Page 7, "species aware models performed slightly better": Could this be due to the proxy species issue mentioned above?
- Figure 2A: Please provide p-values for the statistical significance of the improvement of language models over the baseline approaches.
- Figure 2B: What is the source of the "curated PWM"?
- Figure 3B: What exactly is being plotted here? The probability of the LM generating the full sequence TGTAAATA? Or the product of likelihoods across all individual nucleotide positions? Where are two two blue points? I do not see them. Also, two "instances" of what?
- Figure 3B, "notably degraded": Please provide p-value and state which test was used.
- Figure 3D caption, "better reconstructed": Same request to provide p-value and test.
- Page 10, paragraph on top of page: It would be great if here and elsewhere in the manuscript, the authors could attempt to provide more detailed intuition on how the language models manage to make their predictions, and how combinatorial sequence logic is captured by them, for the benefit of readers who come at this paper from a more biological background (i.e., potential users rather than algorithm developers).
- Figure 4A caption, "notably better reconstructed": Please provide p-value and test.
- Page 11, "Puf3 motif tends to be well reconstructed: Again, a p-value would be nice!
- Figure 5D: Are three of the logos intentionally upside down? If so, why?
- Page 15, section on Masked language modeling: "Why is 10% mutated and 10% left unchanged? I would expect that either a particular position (and all features that depend on it) are masked so that it can be part of the test set, or it is not masked and then it is part of the training set. Why introduce mutations of the actual genome sequence? If there is a valid technical reason for this, better to explain it here and not merely cite Ref. 13.

**Authors’ response to reviewers**

Reviewer #1: Karollus, et al. present an application of large language models (LLMs) to learn features of DNA sequences in non-coding regions across hundreds of yeast genomes. They then investigate how well the LLMs can recover masked sequence examples, particularly focusing on the S. cerevisiae genome that was unseen during training. Remarkably, despite having not been provided any form of regulatory information during training, the LLMs perform better at reconstructing functional regulatory elements such as TF or RBP binding sites. The manuscript thus claims that the LLMs are "capturing" regulatory information within the genome sequences.

Several other publications have applied LLMs to non-coding sequences over the past few years, and the current manuscript does not claim to advance the underlying neural network methodology (it uses the DNABERT LLM model). Rather, the innovations in the current manuscript include how the LLMs have been applied across a large collection of genome sequences. And in particular, this manuscript proposes LLM reconstruction accuracy as a metric for assessing regulatory conservation and innovation across species.

Overall, I think this manuscript presents a powerful application of LLMs to uncover regulatory signals across species. I believe it will be of great interest to the readership of Genome Biology. I have relatively few comments about the manuscript.

1) The results presented in the manuscript are impressive; reconstruction accuracy scores passed into TF-MoDISco enables the detection of TF and RBP binding motifs in S. cerevisiae regulatory regions, and the models appear to learn spatial contexts of some motifs (e.g., higher reconstruction accuracy of the TATA box near TSSs). However, it is difficult to get a sense for how generalizable these results are, or whether we are being presented the cherry-picked best cases. Fortunately, an almost-complete set of S. cerevisiae TF binding sites have been characterized in high resolution under YPD conditions (Rossi, et al., Nature 2021). While the current manuscript uses some of these ChIP-exo datasets in evaluations, there would appear to be an opportunity to more globally assess sensitivity and specificity of reconstruction accuracy compared with other metrics such as phyloP. Obviously, a caveat would apply to specificity calculations, since non-YPD condition-specific TF binding sites could exist that have not been profiled by Rossi, et al.

We have now performed a more comprehensive analysis of the TF binding sites data (new SFig. 7), which strengthened our previous results. The analysis we do requires a protein to fulfill three conditions: (1) the protein should bind DNA at a sufficient number of locations in YPD (otherwise there is no statistical power to do any analysis), (2) it must have a clear consensus motif to search for (otherwise, it is not clear how to do define the negative set and which reconstructions to compare) and (3) not all instances of the consensus motif should be bound (otherwise there is again no statistical power to do analysis). General regulatory factors like Tbf1, Reb1 and Cbf1 have many binding sites and strong sequence preference, which is why we focused on these for the manuscript.

To provide more global results, we have now selected all proteins in the Rossi dataset which exhibited more than 100 peaks genome-wide and then we removed all proteins which did not have a PWM of at least medium confidence in the YeTFaSCo database (this removes e.g. histones, components of the polymerase, co-factors and similar). This gives 51 TFs.

Over these 51 TFs, the species LM model provides statistically significant discrimination between bound and unbound sites for two thirds, outperforming the phastCons conservation score (Wilcoxon, *P* = 1.3e-6, SFig. 7) by a large margin and the agnostic LM (*P* = 0.011, SFig. 7). This is robust to whether we choose the *K. africana* proxy token or average reconstructions over phylogenetically close species (see response to reviewer 2). Accordingly, the results are generalizable to most sequence-specific TFs which have sufficient binding sites in YPD, although performance on individual TFs will depend on a number of factors including the number of binding sites, quality and entropy of the known motif (e.g. motifs which are highly repetitive work less well because the model will generally reconstruct simple repeats regardless of whether they are of regulatory importance) and whether the model has learned the motif.

2) Providing the species label during training enables the LLMs to more accurately capture species-specific regulatory signals. What do the species labels enable the models to learn? I presume the models learn to vary the oligonucleotide composition changes based on the species label provided, but could this be demonstrated? For example, by examining how "reconstructions" of random sequences vary dependent on the species label.

We have now conducted a deeper exploration of the information learned through the species token (SFig. 10-13) in two ways.

On the one hand, we now show an example where swapping the species token leads to changes in reconstruction which are consistent with literature, which indicates that the token is used by the model to encode evolutionary changes in motif usage (SFig. 13).

On the other hand, we now investigate the species embedding space (SFig. 10-12). We find that the first principal component of the species tokens of the 5’ species LM captures species-specific GC content, in line with the intuition of this reviewer that the model captures

species-specific random sequences biases. Moreover, we observed that the distances between species tokens reflect the known taxonomic relationships of the species in our training set.

3) Could the authors comment on whether adding information about gene homology could also help the LLMs to learn more specific regulatory features? For example, if genes were grouped by homology, could per-group labels be provided during training?

Intuitively, providing gene homology group information could help such a model. However, this opens up many modeling decisions (what notion of homology should be provided, etc). Also this could restrict the applicability of the modeling approach, for instance hindering application on synthetic assays, reporter genes, or on genes with few homologues (e.g. many lncRNAs in

human). Making LMs gene-aware, in addition to species-aware, is a research direction we are now mentioning in the discussion.

Reviewer #2: In this manuscript, the authors explore the utility of DNA language models (specifically, the DNABERT implementation from Ref. 14) for annotating non-coding genomic sequence in terms of where cis-regulatory elements are expected to occur relative to genomic landmarks such as a gene's transcriptional start site or UTRs. According to the authors, it is not yet computationally feasible to do this for mammalian genomes, so they do this for a set of 800 fungal genomes.

This is an interesting and useful study that gives authors a concrete practical sense of what a LM can offer. Predictive performance takes the form of the likelihood of correctly reconstructing the true base identity at masked genomic positions based on knowledge of the actual local genomic sequence context. Since the surrounding sequence contains features that are correlated with spatial landmarks, it is perhaps not surprising that overall, predicted spatial patterns (such as in Figure 3A) are consistent with expectation. The most interesting analysis in my view were those where functionality of regulatory elements was predicted from local sequence context, e.g., whether a given consensus sequence was bound by a TF or not (Figure 3D), or the use of the LM embedding as a predictor in regression mode (Figure 5).

I have one major technical concern that needs to be addressed by the authors in a convincing manner: The popular model yeast Saccharomyes cerevisiae, for which a plethora of functional data is available, plays a special role, in that it is held out from the training set of the LM, so that it can serve as a test set. Now, on page 16 the authors state that in order to use their

species-aware LM to make predictions of regulatory for the species-aware version of their model to S. cerevisiae, they had to select a proxy species "based on overall reconstruction achieved." This sounds to me like a basic violation of the need for strict separation between testing and training set. This is particularly relevant since the authors repeatedly comment that the performance of the species-aware LM relative to the species-agnostic one is "remarkable." Maybe it is remarkable for the wrong reasons, especially when a best-performing proxy species can be chosen from among 800 candidates?

Note that a possible solution to this problem would be to not hold out one entire genome, but to hold out a large number (say, 50%) of S. cerevisiae genes from the training set, while keeping the other 50% as part of the training set, so that the species-specific features can be learned for S. cerevisiae, and the problematic selection of a proxy species is avoided altogether. We have now addressed this point in two-fold. First, we clarify how we selected the species from the training set for inference on the held-out species S. cerevisiae. This was done among the eight phylogenetically closest species and not all 800. Second, we have now proposed two schemes that respect a strict separation between training and test. One is the scheme proposed by this reviewer. The other scheme averages predictions using a set of phylogenetically close

species. We have performed extensive analyses comparing those approaches. These results are part of a new supplementary information section and new supplementary figures (SFig.

14-23). In brief, we found that averaging predictions using a set of close species is the most robust approach. However, all three approaches (single close species, averaging across close species and learning a token on half of the genome as suggested) perform similarly well: up to a few individual motifs all the results hold.

Further comments:

- Page 3, "after fine-tuning": Can you give some intuition what this means in practice? In general, it would be great if the authors would not assume that the readers are already intimately familiar with language models, and provide some more detailed explanation and intuition of how these models work. The diagrams in Figure 1C and 1D are not as informative as they could be perhaps.

We have explained the term fine-tuning and have attempted to make the figure more informative. We have improved Figure 1D by displaying more sequences to emphasize that each model is trained on sequences of 800 fungi on all genes jointly, and added information about the total number of sequences.

We have decided to keep Figure 1C as it was. Figure 1C highlights the difference of our modeling approach compared to previous DNA language models. The internal details (transformer architecture) of the model mostly correspond to the previous literature and are better described elsewhere (DNABERT, Ji et al., 2021), which now also refer to in the legend of Fig. 1C.

- Page 5, "the need and potential for alignment-free approaches": Alignment-free approaches have been used in the past to analyze non-coding genomic DNA. See for instance PMID 17993685, 16809671, 18586710. It may be useful to cite some of that older work here. There are also older methods akin to language models that have been applied to find regulatory elements based on a statistical analysis non-coding sequence alone (e.g., PMID 10944202) that the readers of this manuscript may find interesting.

We thank the reviewer for helping to connect our work to previous approaches. We have added citations to these works and related ones (Gordan et al. 2010, Zielezinski et al. 2017) in the first result section. We have also introduced a short note on how language models compare to these approaches. We hope that this section also helps to clarify how language models can learn motif dependencies in a more flexible fashion than methods based on earlier modeling paradigms.

- When building the LM, were all genes analyzed jointly, or was the LM build separately for all orthologs of the regulatory region of each individual gene? It is not 100% clear from the text, so define this more explicitly. Could it be useful for build per-gene LM's?

We have now clarified in the first results section that models are trained on all genes jointly and no orthology information is provided to the model. We expect that training one LM per gene would likely not work with our architecture, since we would only have around 800 sequences to learn 100 million parameters. Moreover, we expect that many features of the regulatory code are shared across genes, so training jointly should lead to better generalization. However, providing information about orthology or gene function could be a way to improve the model (see also response to Reviewer 1).

- Page 7, "we were able to recover some of these motifs de novo": So are these cherry-picked examples? To counter this impression, it would be good if the success rate across all known consensus motifs could be reported here, and how this rate compares to that of a reference "standard" approach.

We now provide all Modisco hits for the 5’ and 3’ regions and compare those to motifs found using STREME as a reference approach. We note that our Modisco analysis is mainly intended as a technique to interrogate what the model has learnt, not as a general purpose motif finding tool. Thus it gives no explicit statistical guarantees, e.g. p-values for motifs. Nevertheless, we find that both methods result in similar amounts of hits which can be mapped to known motifs.

Specifically, with Modisco we find 31 hits for 3’ regions, of which we could match 6 (~19%) to known motifs and 12 are easily identified as simple repeats. For the 5’ regions, we get 51 hits, whereby 18 (~35%) correspond to known motifs and 13 correspond to simple repeats.

With STREME, we find 15 hits in 3’ regions (using default settings except that we use RNA mode and a minimum width of 5, as the Whi3 motif is only five nucleotides long), whereby 3 (~20%) correspond to known motifs and 5 correspond to simple repeats. For the 5’ regions, STREME (default settings) get 39 hits, whereby 9 (~23%) correspond to known motifs and 5 correspond to simple repeats.

- Page 7, "species aware models performed slightly better": Could this be due to the proxy species issue mentioned above?

As explained above and in the new supplementary information section, the species-aware model outperforms the species-agnostic one also if we average over phylogenetically close species.

- Figure 2A: Please provide p-values for the statistical significance of the improvement of language models over the baseline approaches.

We now indicate in Figure 2A whenever the species-aware LM significantly outperforms (P < 0.05, binomial test) the strongest baseline for each motif.

- Figure 2B: What is the source of the "curated PWM"?

We added the citation to the YeTFaSCo database.

- Figure 3B: What exactly is being plotted here? The probability of the LM generating the full sequence TGTAAATA? Or the product of likelihoods across all individual nucleotide positions? Where are two two blue points? I do not see them. Also, two "instances" of what? It is the average of the log-likelihoods across each individual nucleotide, which corresponds to the log of the product of the likelihoods normalized for sequence length. This makes the axis

scale comparable between motifs of different length. We have clarified this in the figure caption and methods section.

One blue dot was erroneously missing. We now use fatter circles and clarified that “instances” refers to instances of the motif in the legend.

- Figure 3B, "notably degraded": Please provide p-value and state which test was used. We have now provided the requested P-value in the figure caption and stated we use Mann-Whitney U test.
- Figure 3D caption, "better reconstructed": Same request to provide p-value and test. We have now provided the requested P-value in the figure caption and stated we use Mann-Whitney U test.
- Page 10, paragraph on top of page: It would be great if here and elsewhere in the manuscript, the authors could attempt to provide more detailed intuition on how the language models manage to make their predictions, and how combinatorial sequence logic is captured by them, for the benefit of readers who come at this paper from a more biological background (i.e., potential users rather than algorithm developers).

We hope the added paragraph which compares language models to k-mer enrichment methods provides some intuition on how these models capture dependencies. We also now refer to the attention mechanism of transformers in the interpretation of the Figure 3 results page 10. This being said, the downside of the flexibility of deep models is that it generally is very difficult to determine exactly how they learn something. Interpretation of deep models is an open area of research and very difficult. As a result we are unfortunately unable to give a fully satisfying answer here.

- Figure 4A caption, "notably better reconstructed": Please provide p-value and test.

We have indicated with a star whenever the difference in reconstruction between motif and remaining nucleotides is significant (P < 0.05, Mann-Whitney U). This is the case for all species except N. crassa.

- Page 11, "Puf3 motif tends to be well reconstructed: Again, a p-value would be nice! If we are not mistaken, this also refers to Figure 4A.
- Figure 5D: Are three of the logos intentionally upside down? If so, why?

Yes, this is because the motif has a negative effect on the prediction (half-life), as conventionally done for Modisco analyses. We have now clarified this in the caption

- Page 15, section on Masked language modeling: "Why is 10% mutated and 10% left unchanged? I would expect that either a particular position (and all features that depend on it) are masked so that it can be part of the test set, or it is not masked and then it is part of the training set. Why introduce mutations of the actual genome sequence? If there is a valid technical reason for this, better to explain it here and not merely cite Ref. 13.

We have now added some explanation to this statement. The original BERT study (Devlin et al., 2018), a model developed for natural language processing, showed that on top masking words, replacing original words with random words improves the representation for downstream tasks. The same approach was later used in DNABERT (Ji et al., 2021). For compatibility, we have used the same procedure. Investigations of the utility of mutated versus masked nucleotides and related training strategy decisions (including length of the k-mer tokens, fraction of mutation and masking etc.) are probably valuable but are out

**Second round of review**

**Reviewer 1**

The authors have appropriately addressed all of my previous comments.

**Reviewer 2**

The authors have addressed all issues raised in my original review in a satisfactory manner. I have no further comments.
